# Supplementary figures and images for: IDH1 regulates human erythropoiesis by eliciting chromatin state reprogramming
Source: eLife. 2025 Apr 29;13:RP100406. doi: 10.7554/eLife.100406 (PMC12040319; doi:10.7554/eLife.100406)

### Figure S1A

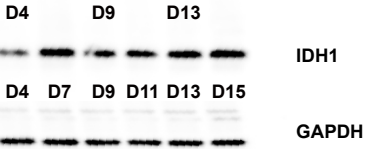

Supplement: Figure 1—figure supplement 1—source data 1. [file elife-100406-fig1-figsupp1-data1.pdf]

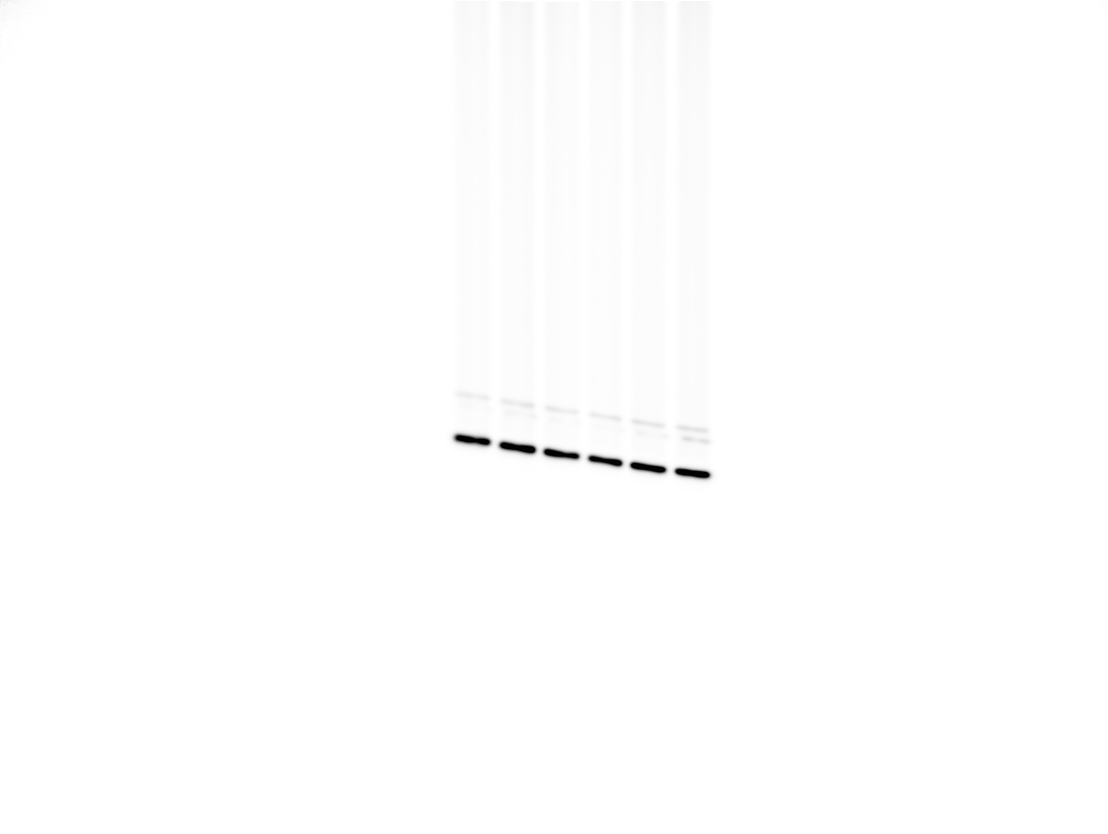

Supplement: Figure 1—figure supplement 1—source data 2. [file elife-100406-fig1-figsupp1-data2.zip › Figure S1-source data 2/SF1-GAPDH.tif]

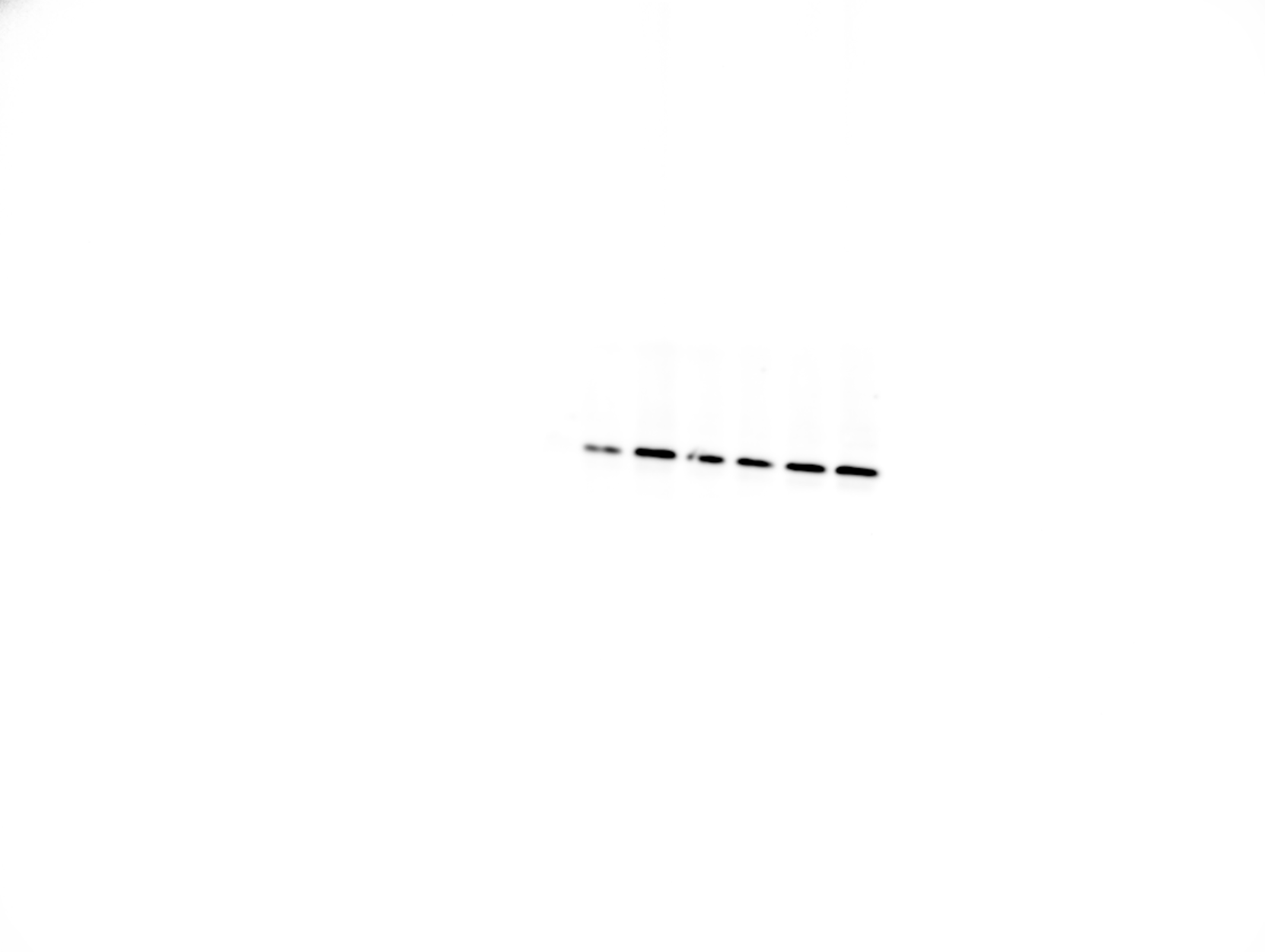

Supplement: Figure 1—figure supplement 1—source data 2. [file elife-100406-fig1-figsupp1-data2.zip › Figure S1-source data 2/SF1-IDH1.tif]

**Figure S2B**

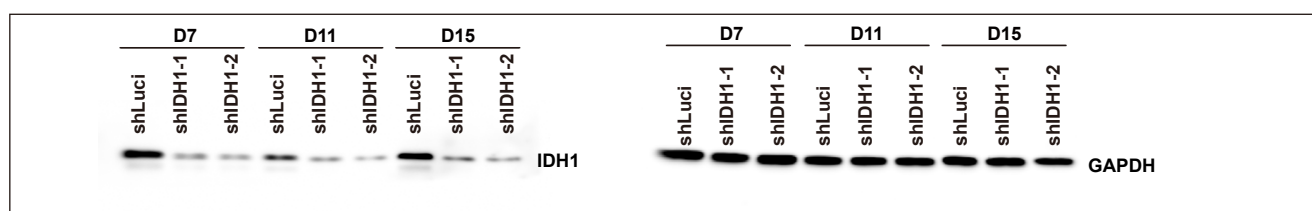

Supplement: Figure 1—figure supplement 2—source data 1. [file elife-100406-fig1-figsupp2-data1.pdf]

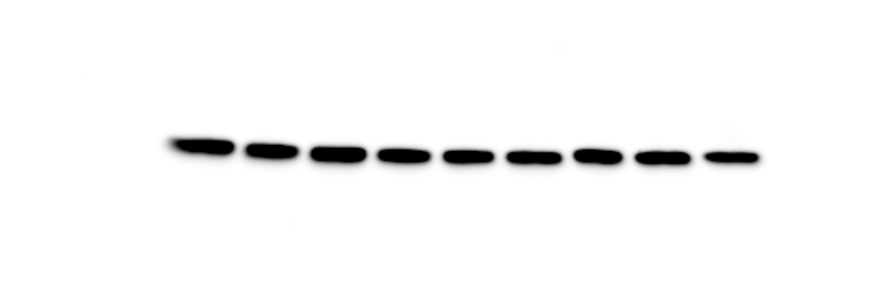

Supplement: Figure 1—figure supplement 2—source data 2. [file elife-100406-fig1-figsupp2-data2.zip › Figure S2-source data 2/SF2-B-GAPDH.tif]

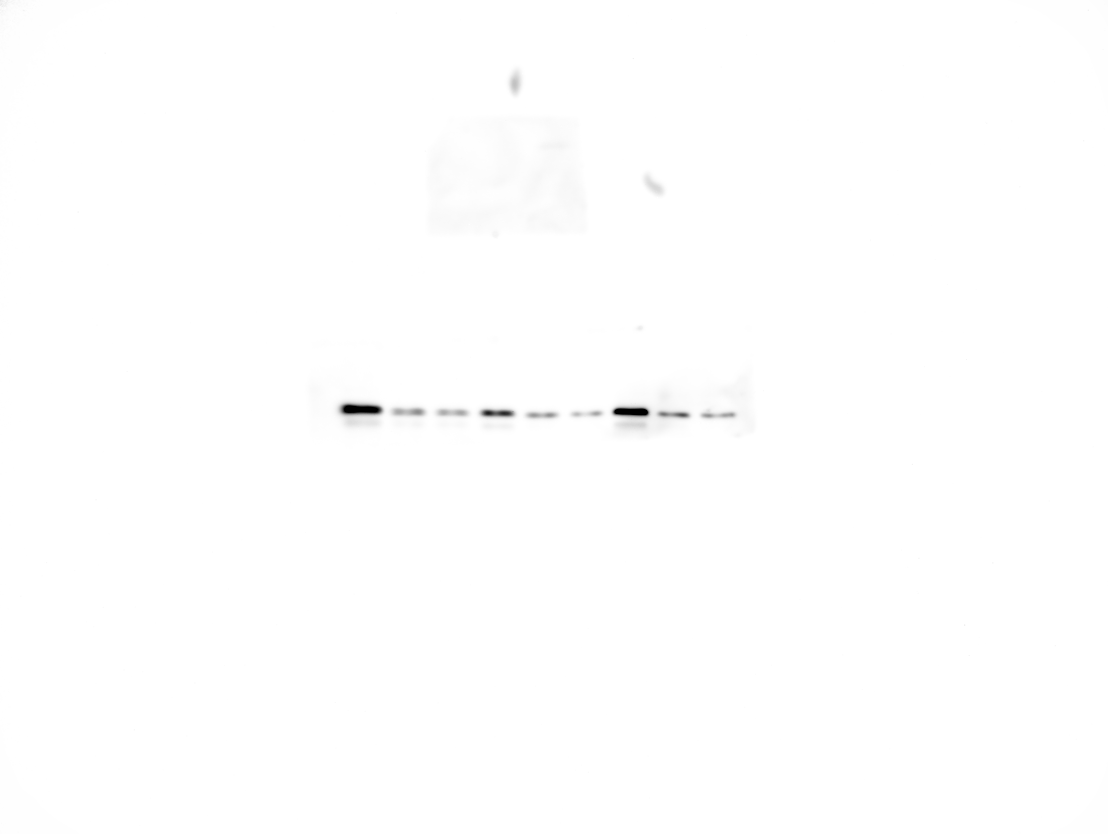

Supplement: Figure 1—figure supplement 2—source data 2. [file elife-100406-fig1-figsupp2-data2.zip › Figure S2-source data 2/SF2-B-IDH1.tif]

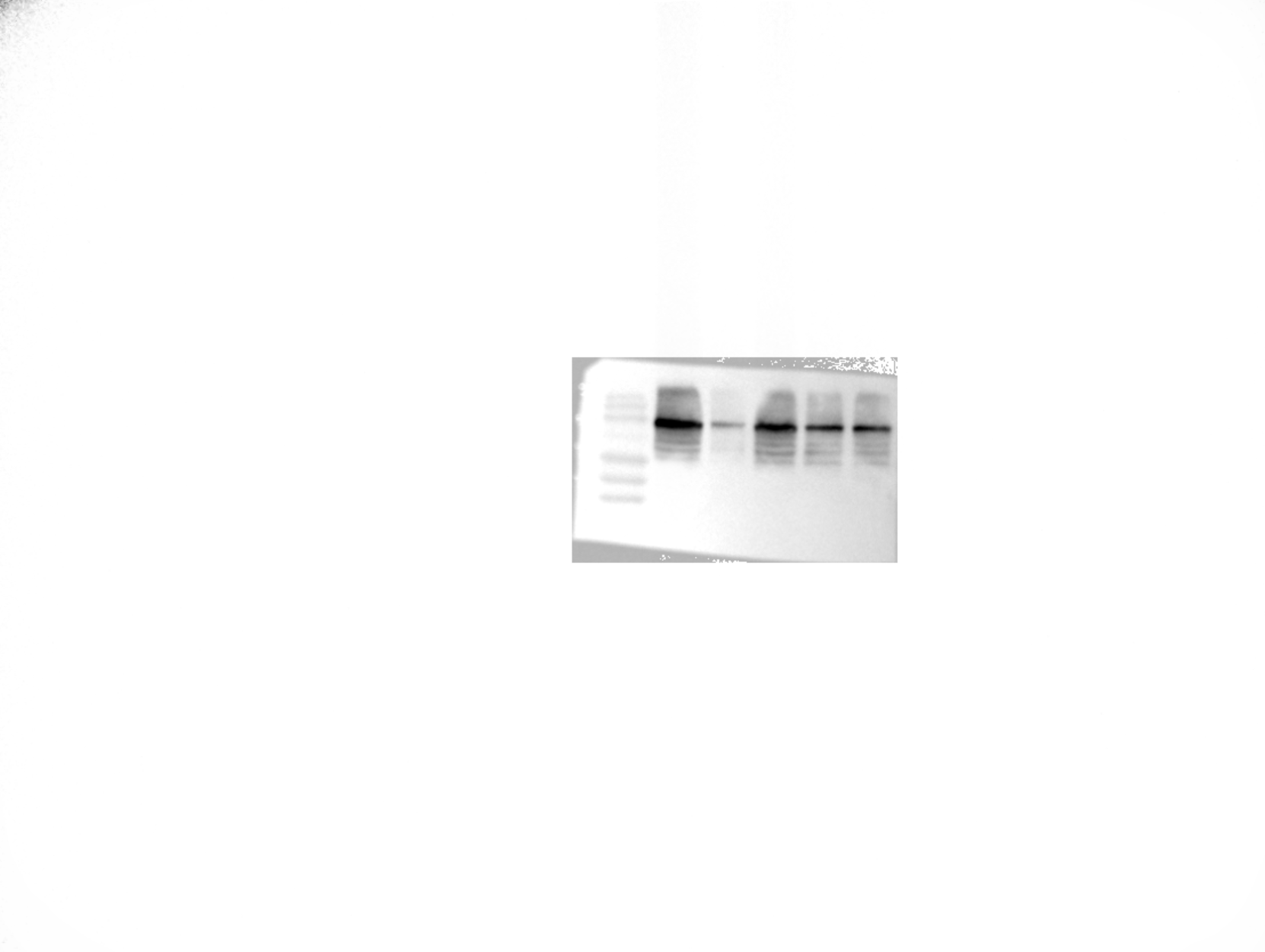

Supplement: Figure 1—figure supplement 3—source data 2. [file elife-100406-fig1-figsupp3-data2.zip › Figure S3-source data 2/2021-0319-163738_pub.tif]

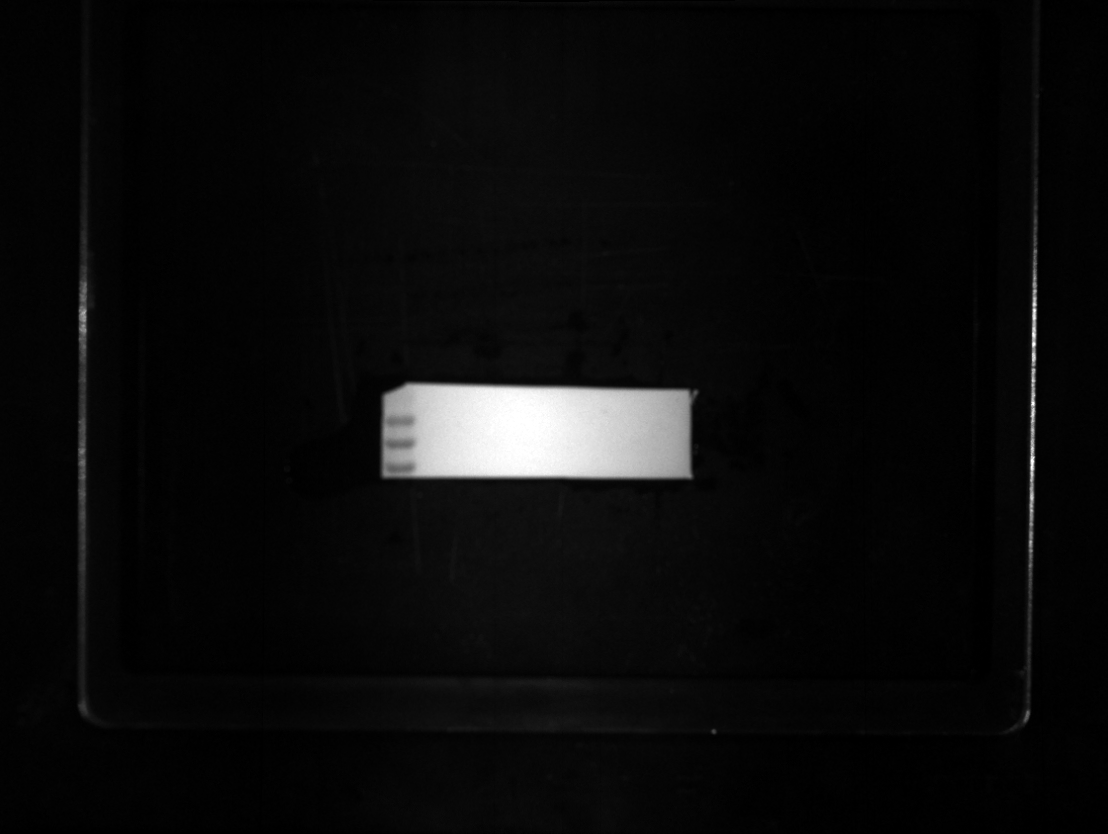

Supplement: Figure 1—figure supplement 3—source data 2. [file elife-100406-fig1-figsupp3-data2.zip › Figure S3-source data 2/2021-0624-152900.tif]

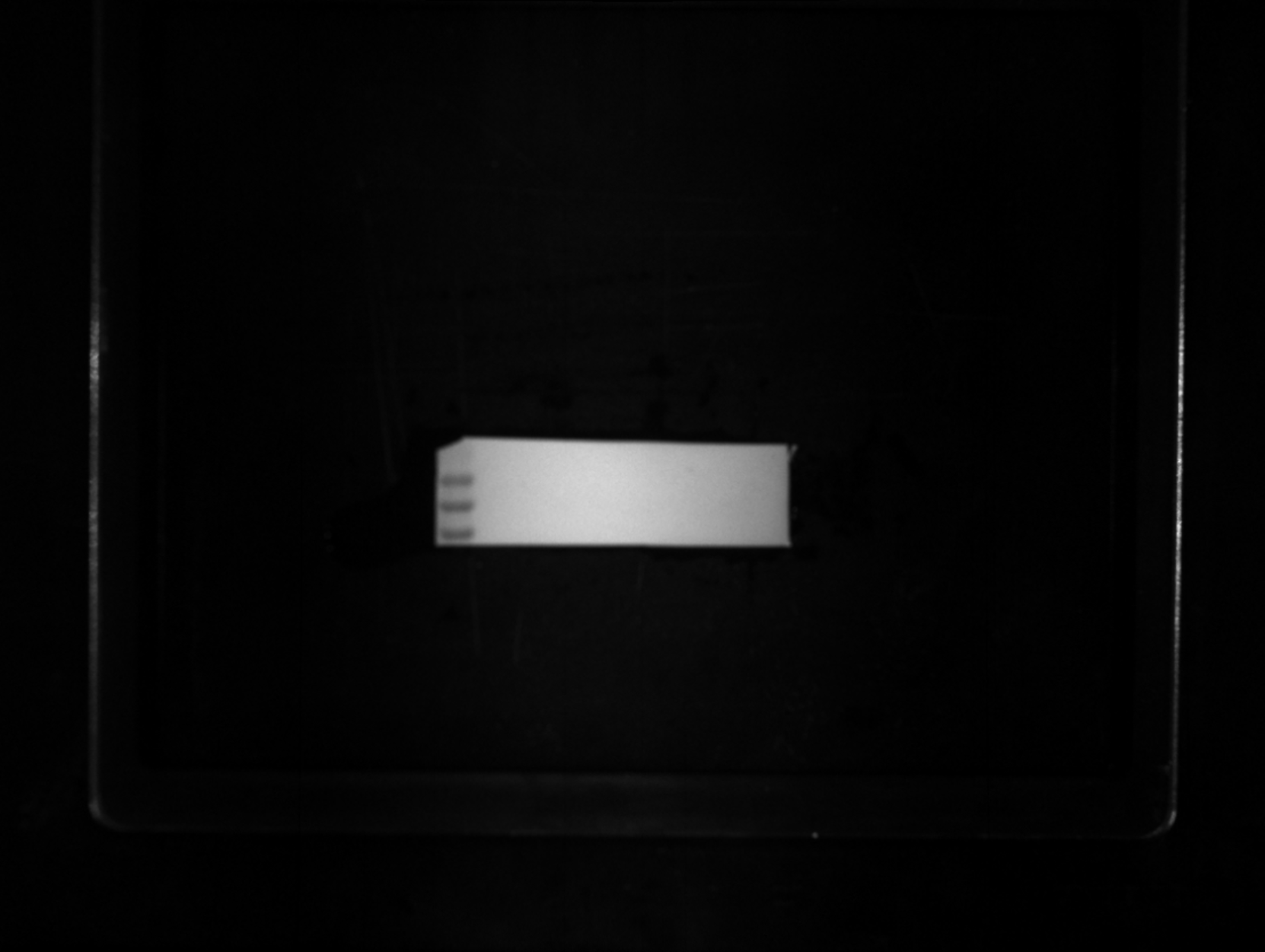

Supplement: Figure 1—figure supplement 3—source data 2. [file elife-100406-fig1-figsupp3-data2.zip › Figure S3-source data 2/2021-0624-152900_pub.tif]

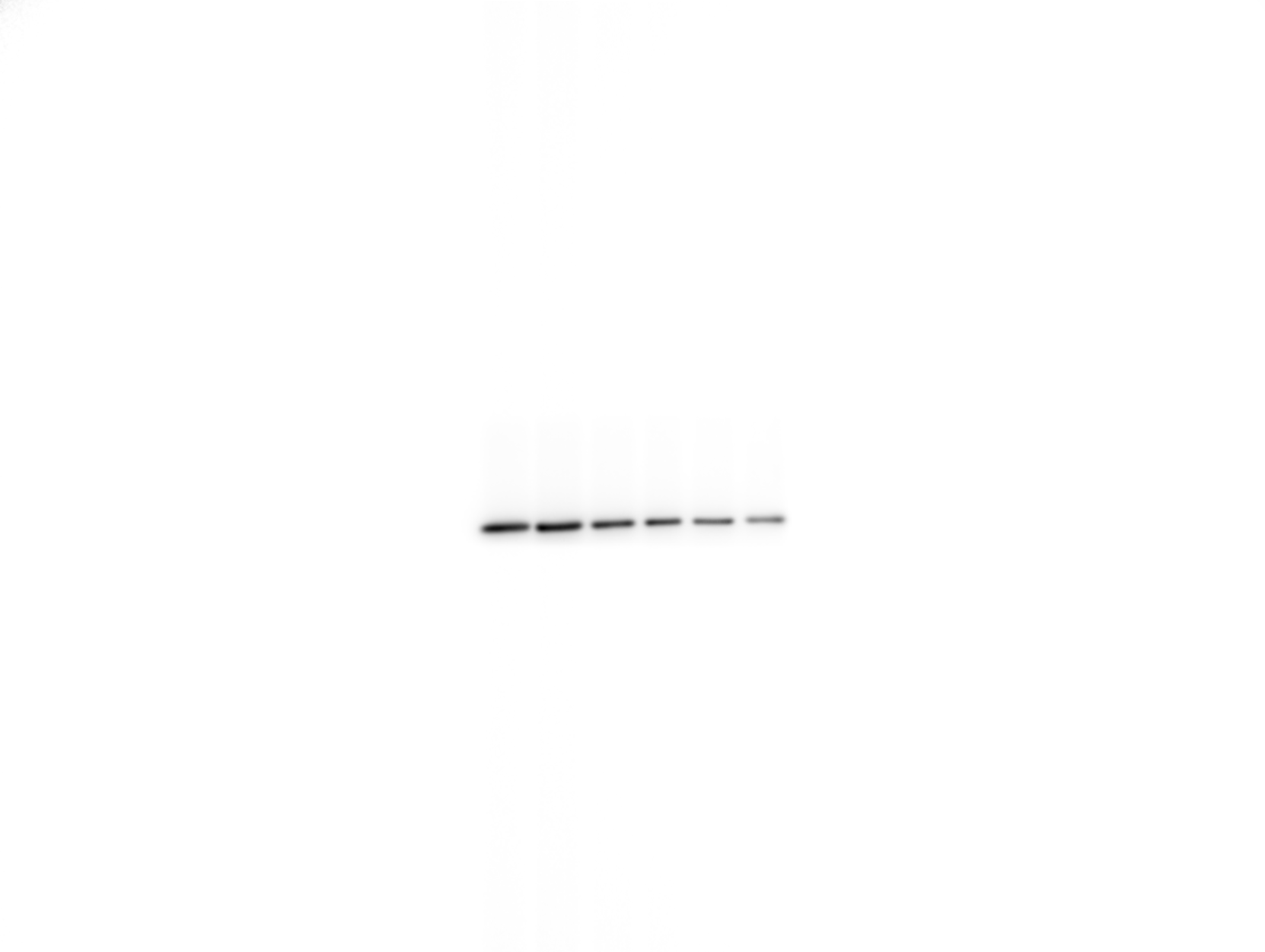

Supplement: Figure 1—figure supplement 3—source data 2. [file elife-100406-fig1-figsupp3-data2.zip › Figure S3-source data 2/2021-0624-152902_pub.tif]

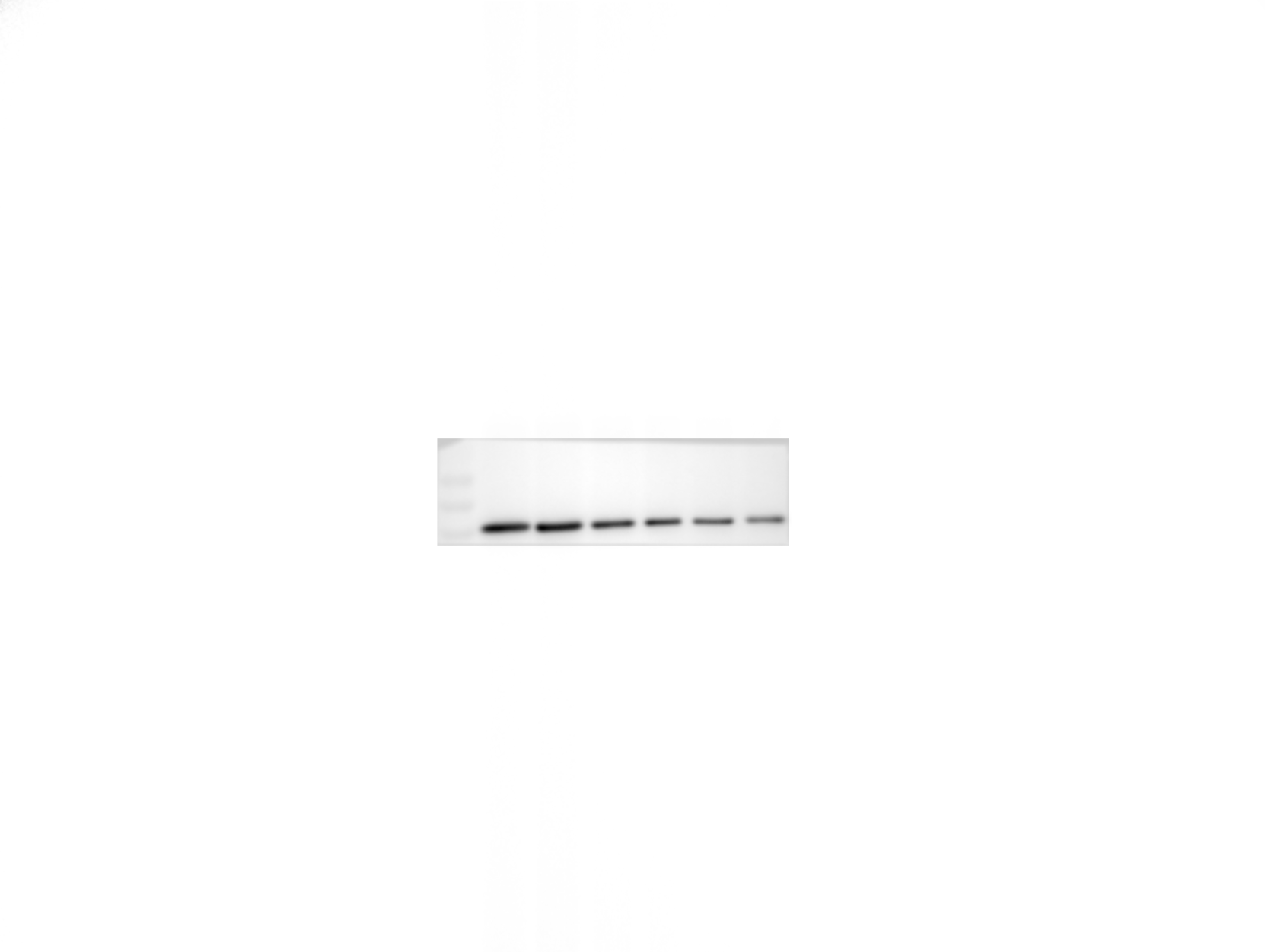

Supplement: Figure 1—figure supplement 3—source data 2. [file elife-100406-fig1-figsupp3-data2.zip › Figure S3-source data 2/2021-0624-152904_pub.tif]

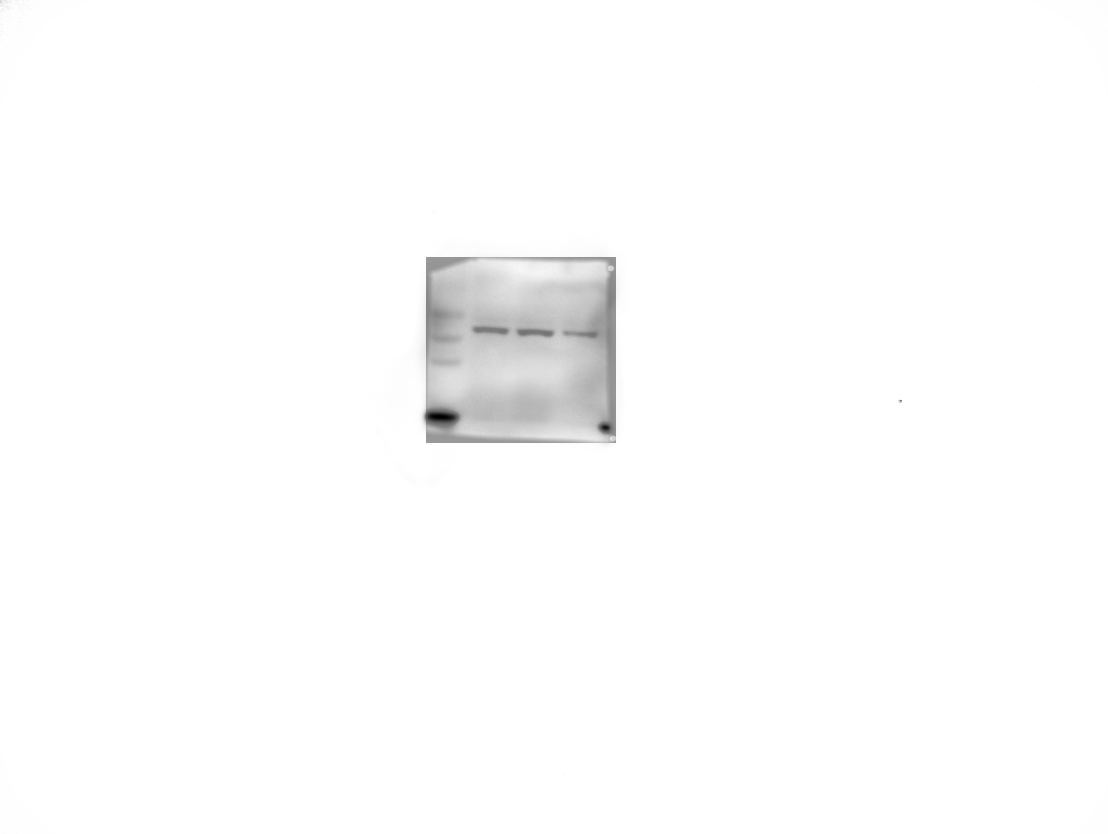

Supplement: Figure 1—figure supplement 3—source data 2. [file elife-100406-fig1-figsupp3-data2.zip › Figure S3-source data 2/S3F1-0630-210242.tif]

Figure 2F

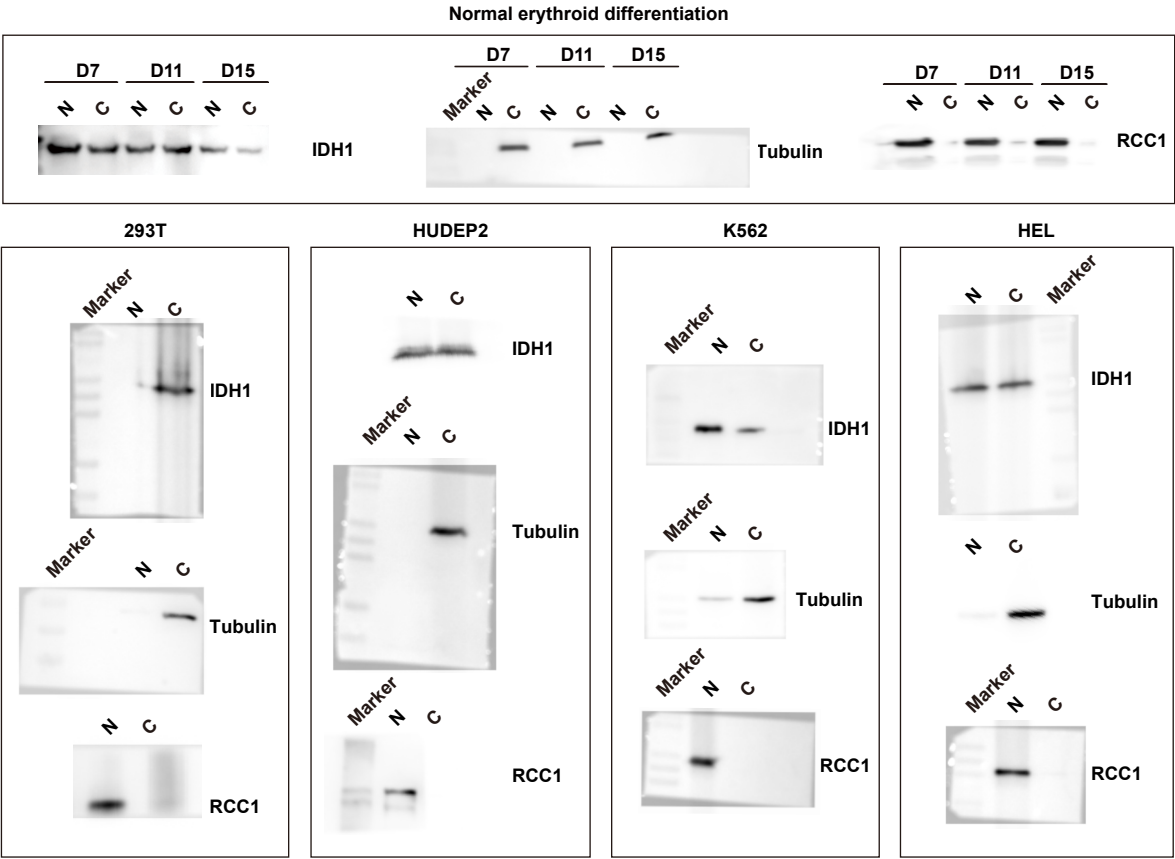

Supplement: Figure 2—source data 1. [file elife-100406-fig2-data1.pdf]

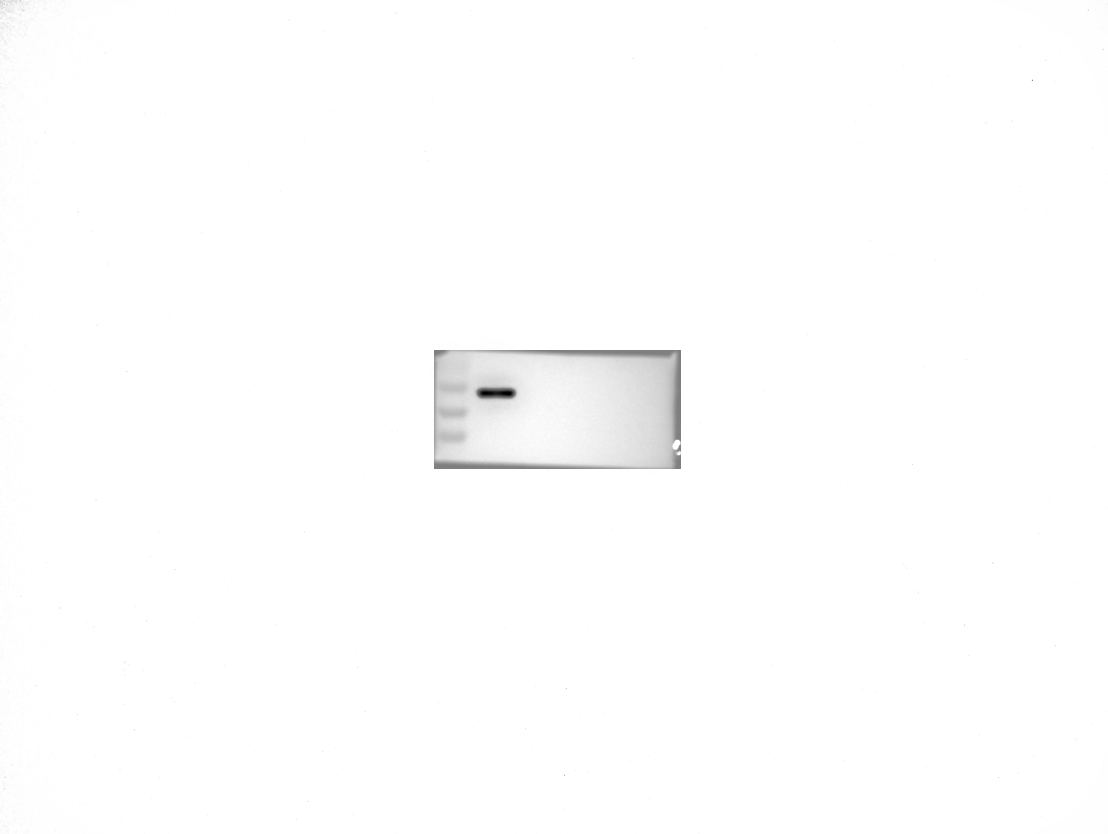

Supplement: Figure 2—source data 2. [file elife-100406-fig2-data2.zip › Figure 2-source data 2/1.tif]

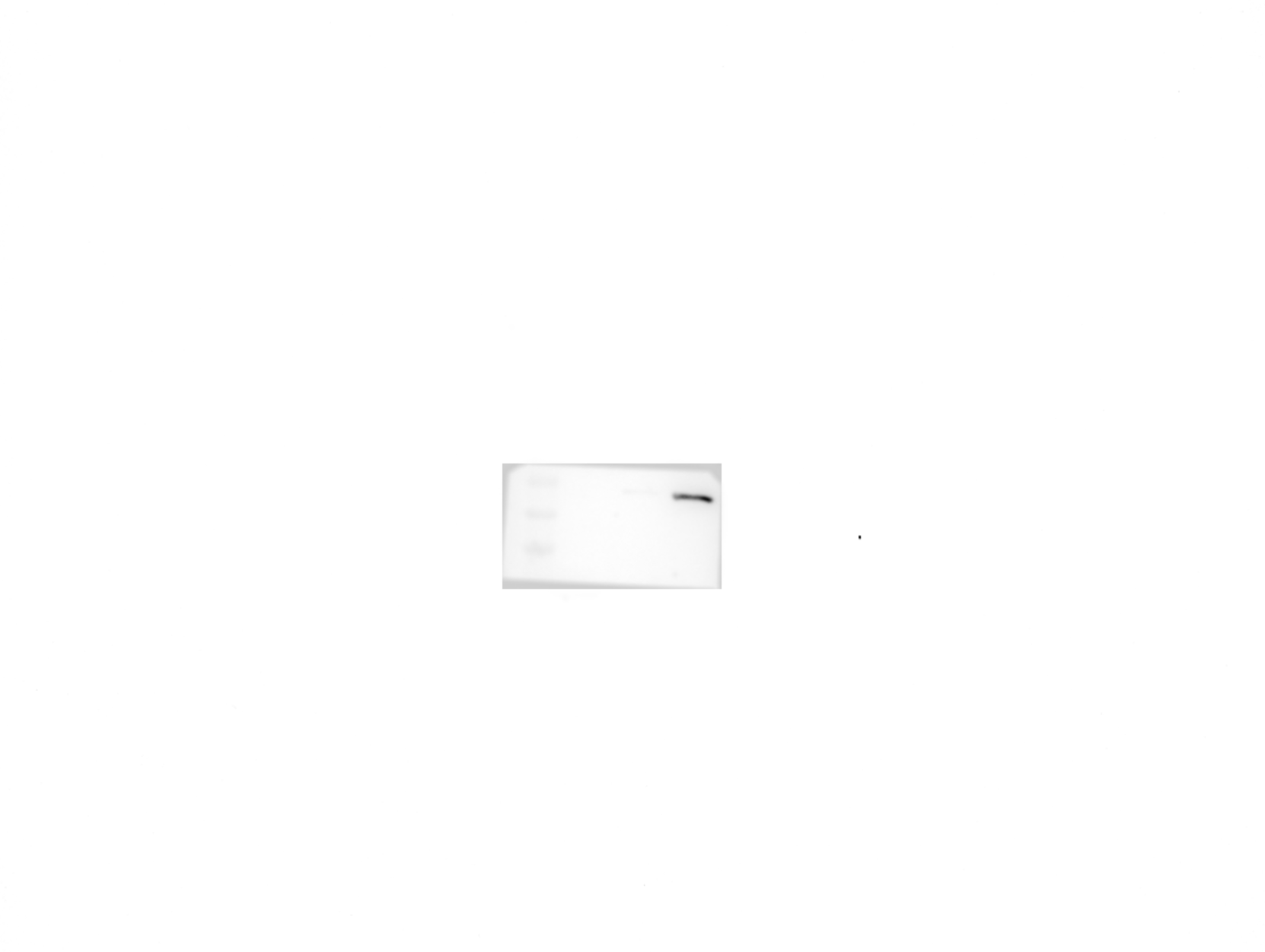

Supplement: Figure 2—source data 2. [file elife-100406-fig2-data2.zip › Figure 2-source data 2/F2-293T-Tubulin.tif]

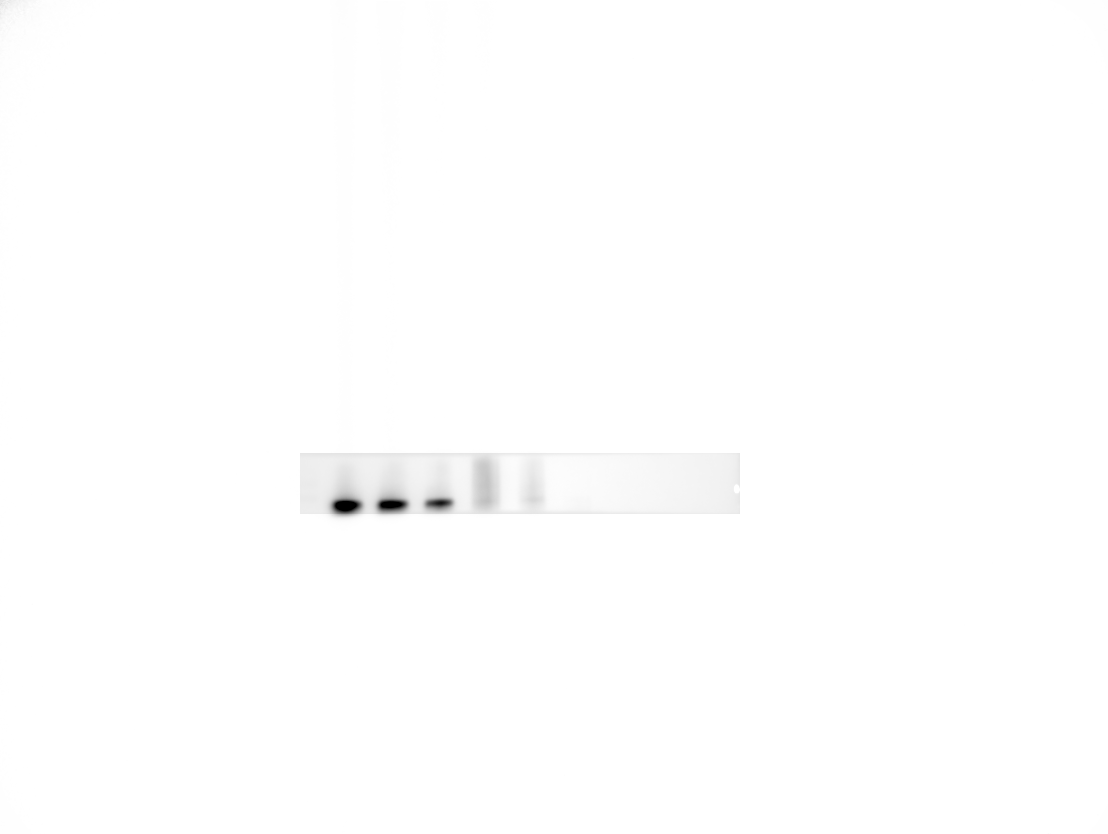

Supplement: Figure 2—source data 2. [file elife-100406-fig2-data2.zip › Figure 2-source data 2/F2-F-293-RCC1.tif]

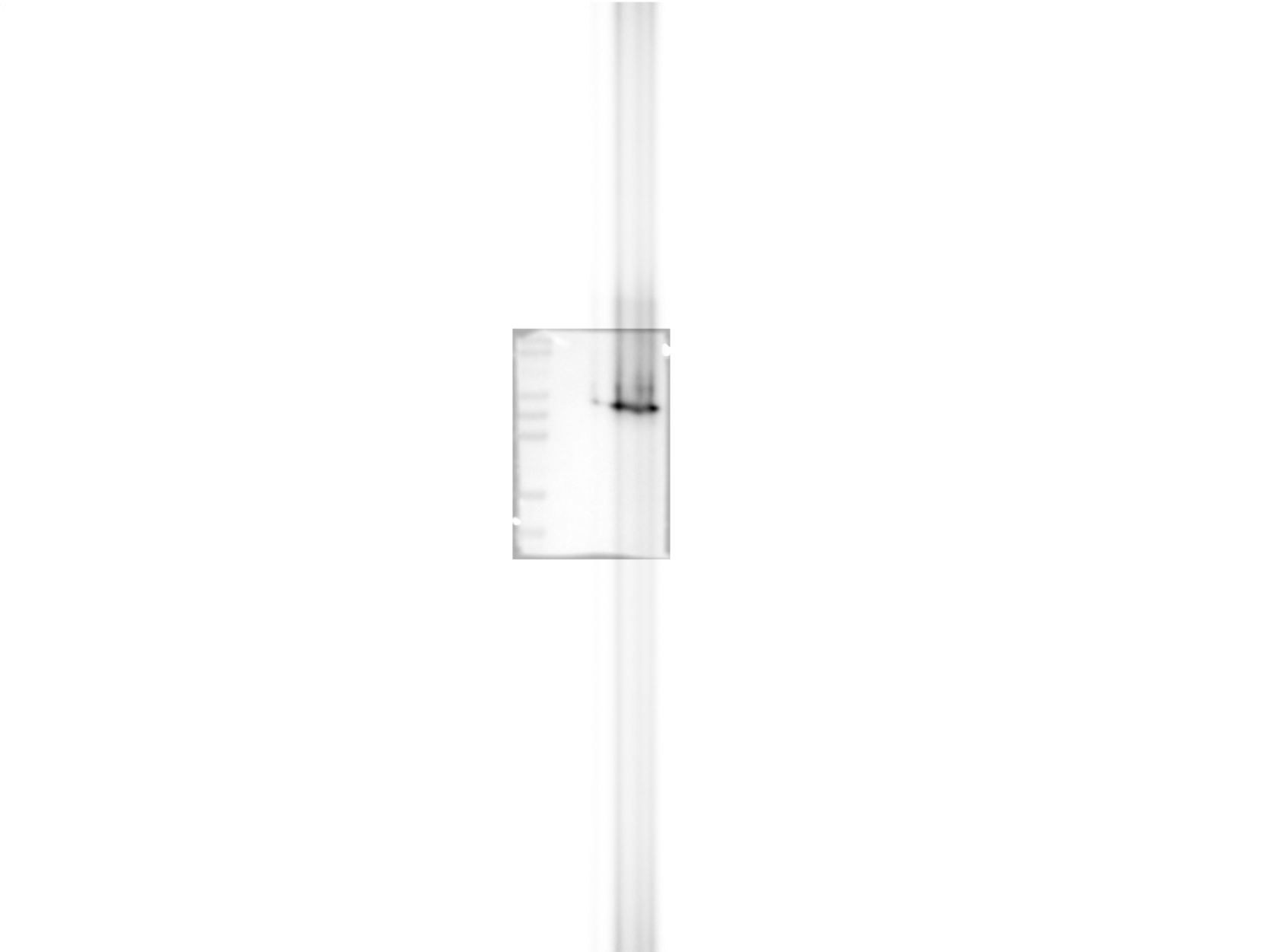

Supplement: Figure 2—source data 2. [file elife-100406-fig2-data2.zip › Figure 2-source data 2/F2-F-293T-IDH1.tif]

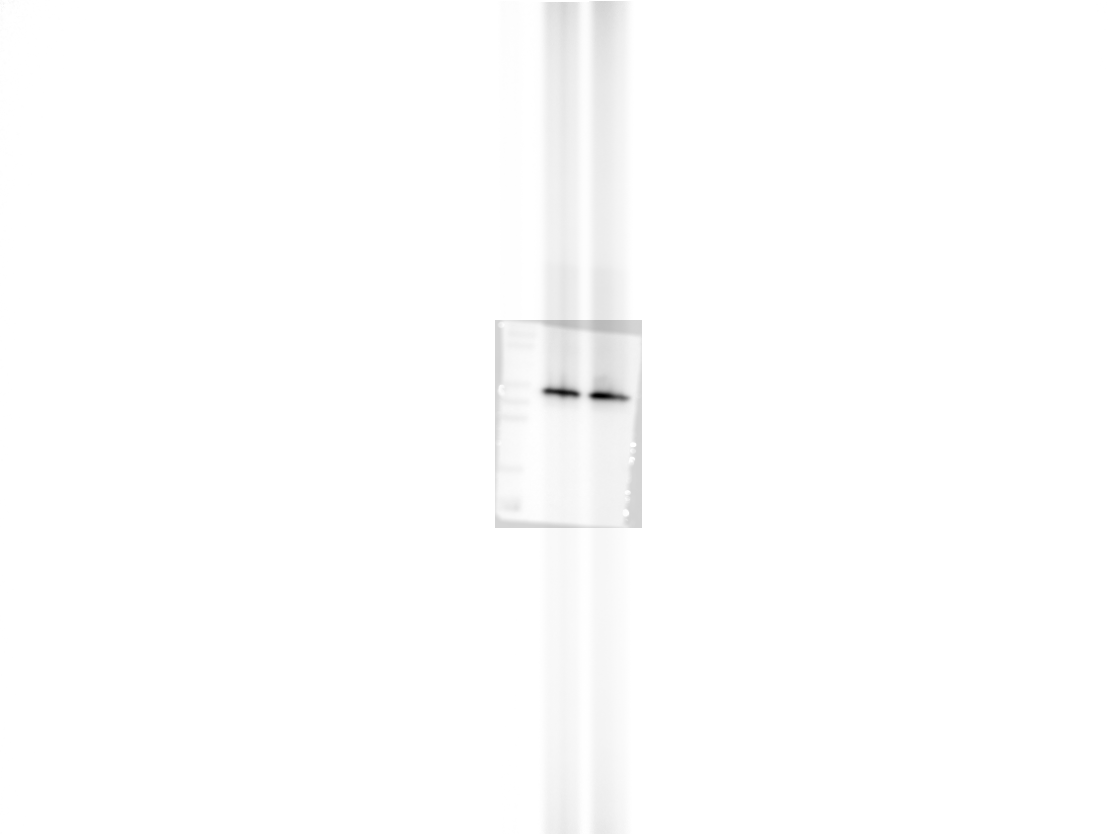

Supplement: Figure 2—source data 2. [file elife-100406-fig2-data2.zip › Figure 2-source data 2/F2-F-HEL-IDH1.tif]

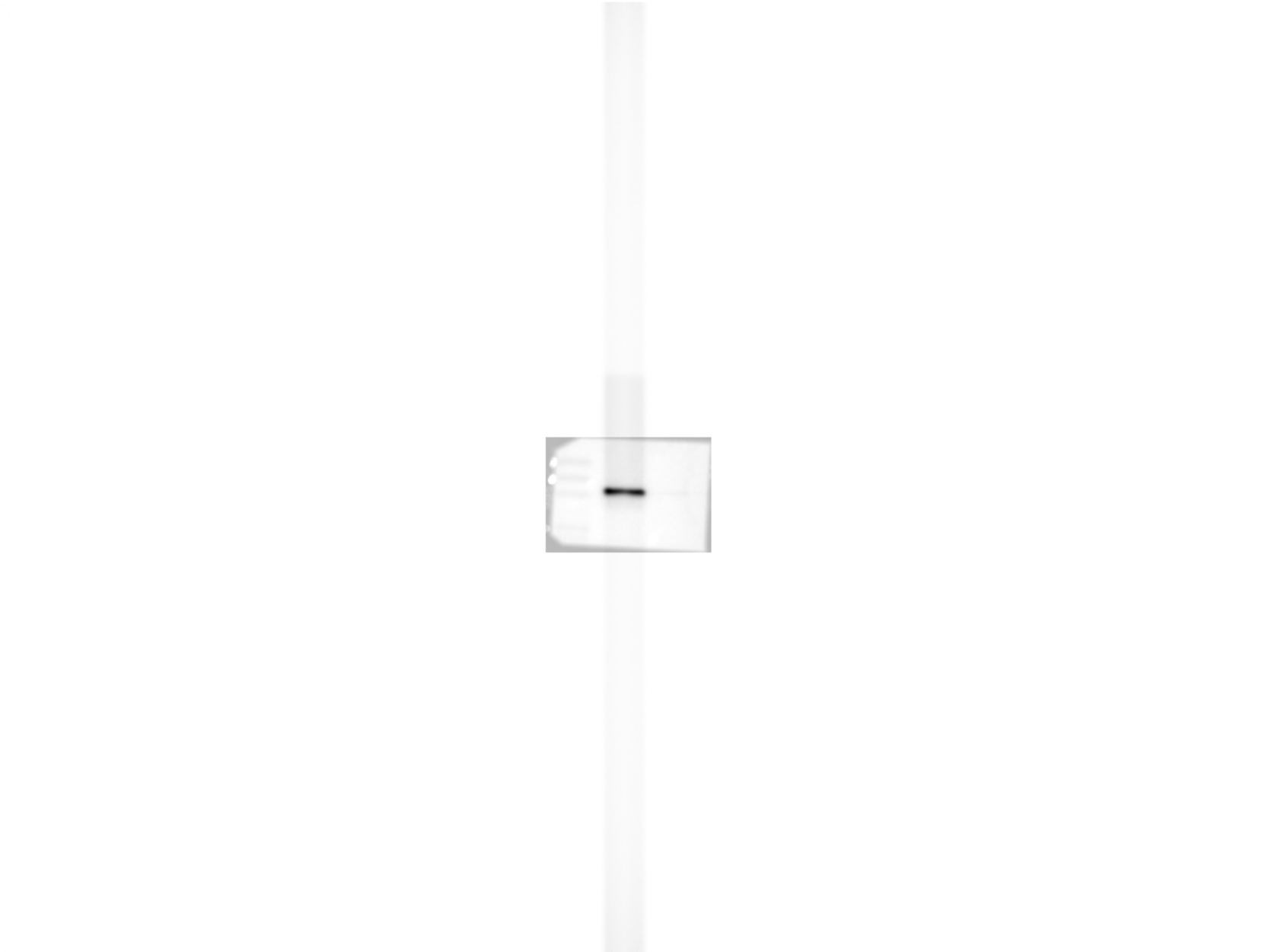

Supplement: Figure 2—source data 2. [file elife-100406-fig2-data2.zip › Figure 2-source data 2/F2-F-HEL-RCC1.tif]

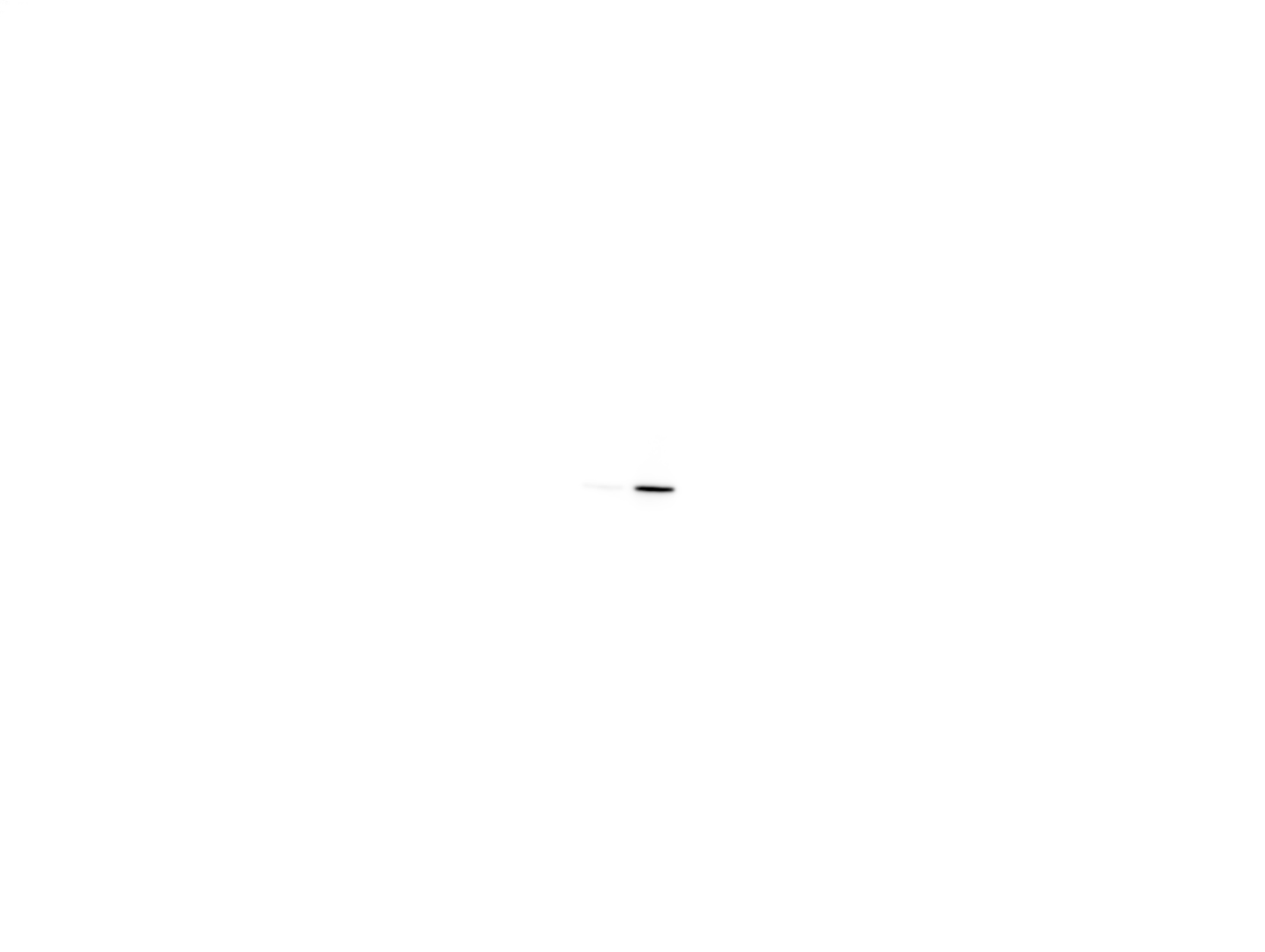

Supplement: Figure 2—source data 2. [file elife-100406-fig2-data2.zip › Figure 2-source data 2/F2-F-HEL-Tubulin.tif]

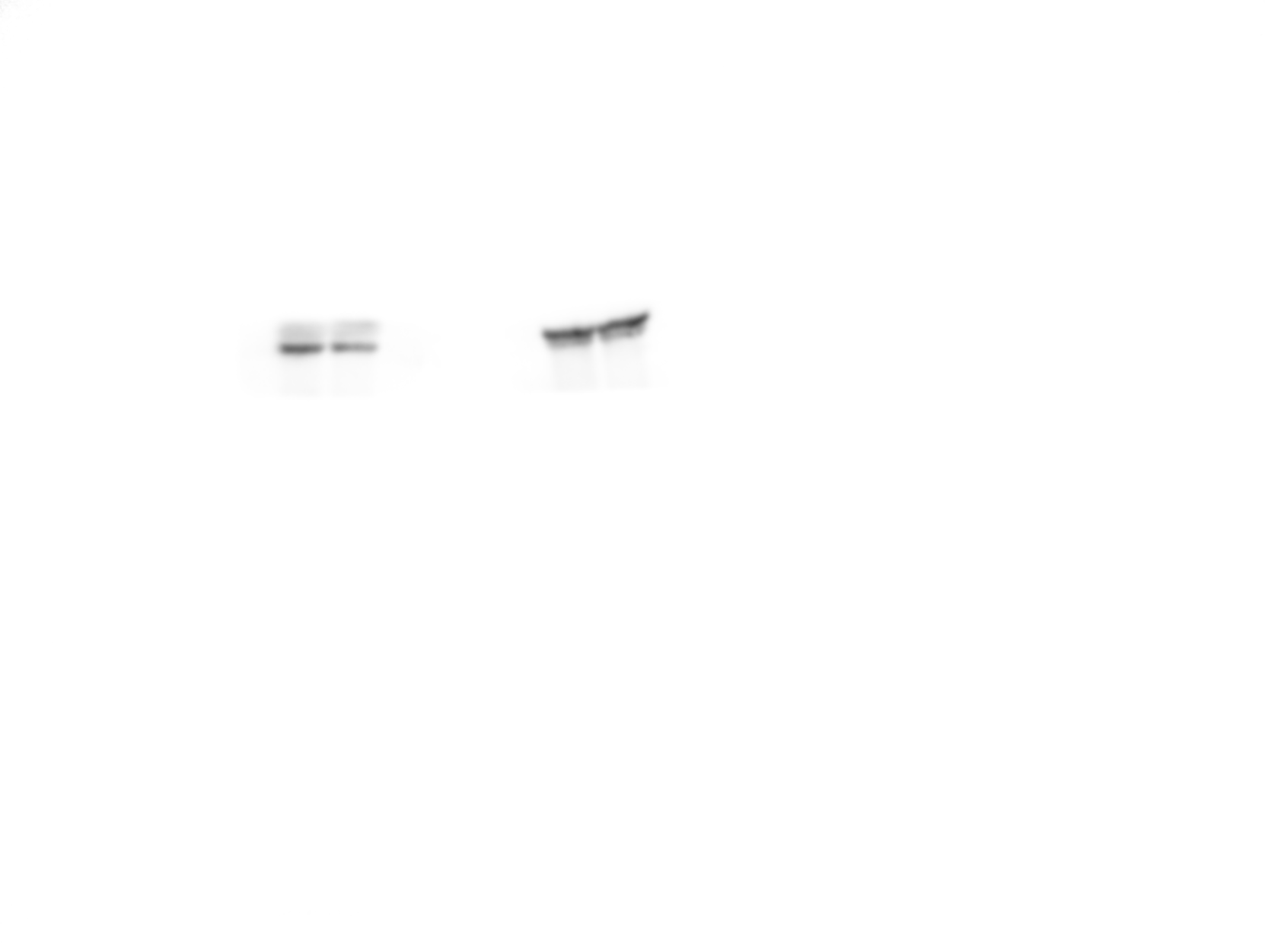

Supplement: Figure 2—source data 2. [file elife-100406-fig2-data2.zip › Figure 2-source data 2/F2-F-HUDEP2-IDH1.tif]

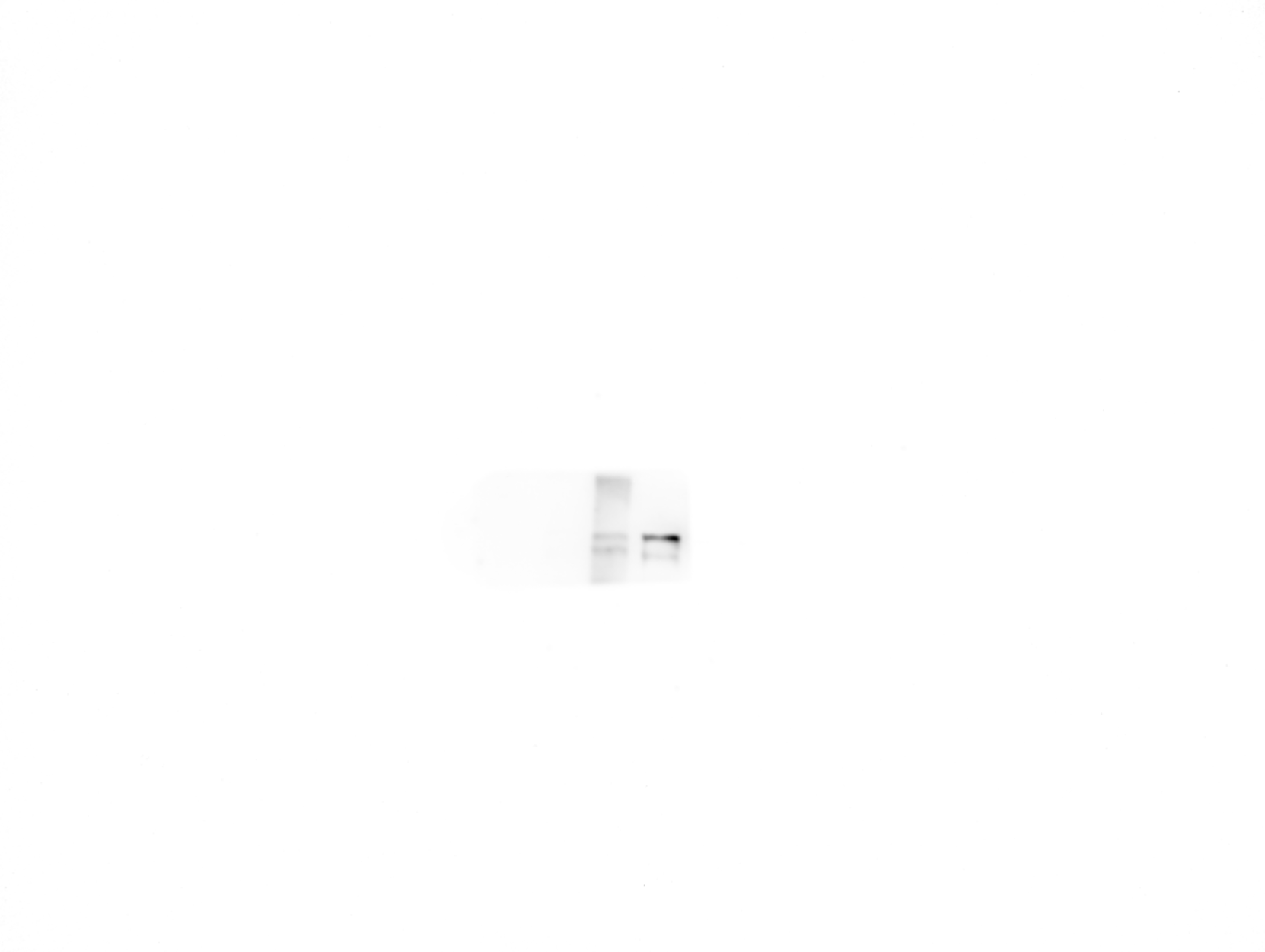

Supplement: Figure 2—source data 2. [file elife-100406-fig2-data2.zip › Figure 2-source data 2/F2-F-HUDEP2-RCC1.tif]

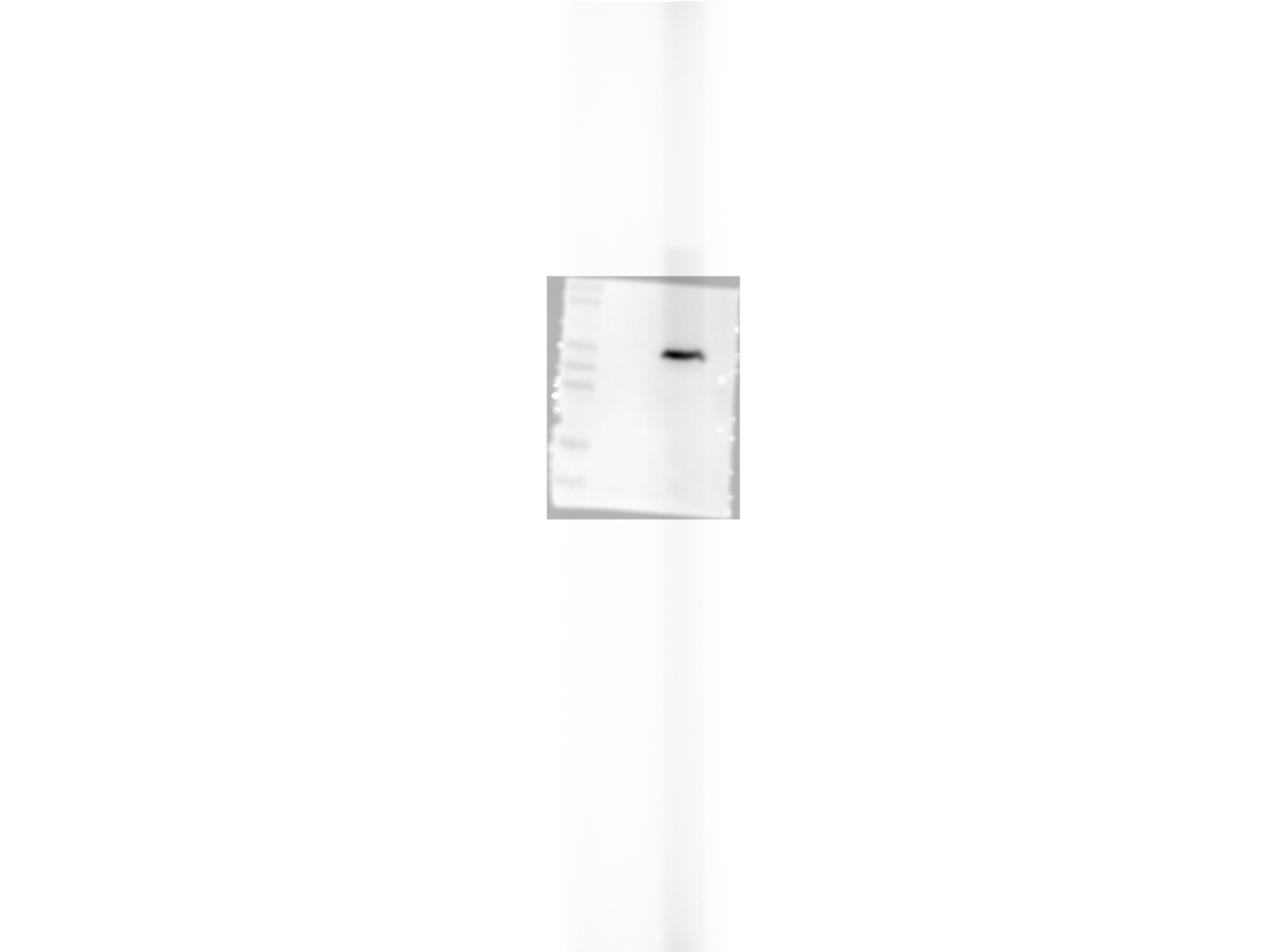

Supplement: Figure 2—source data 2. [file elife-100406-fig2-data2.zip › Figure 2-source data 2/F2-F-HUDEP2-Tubulin.tif]

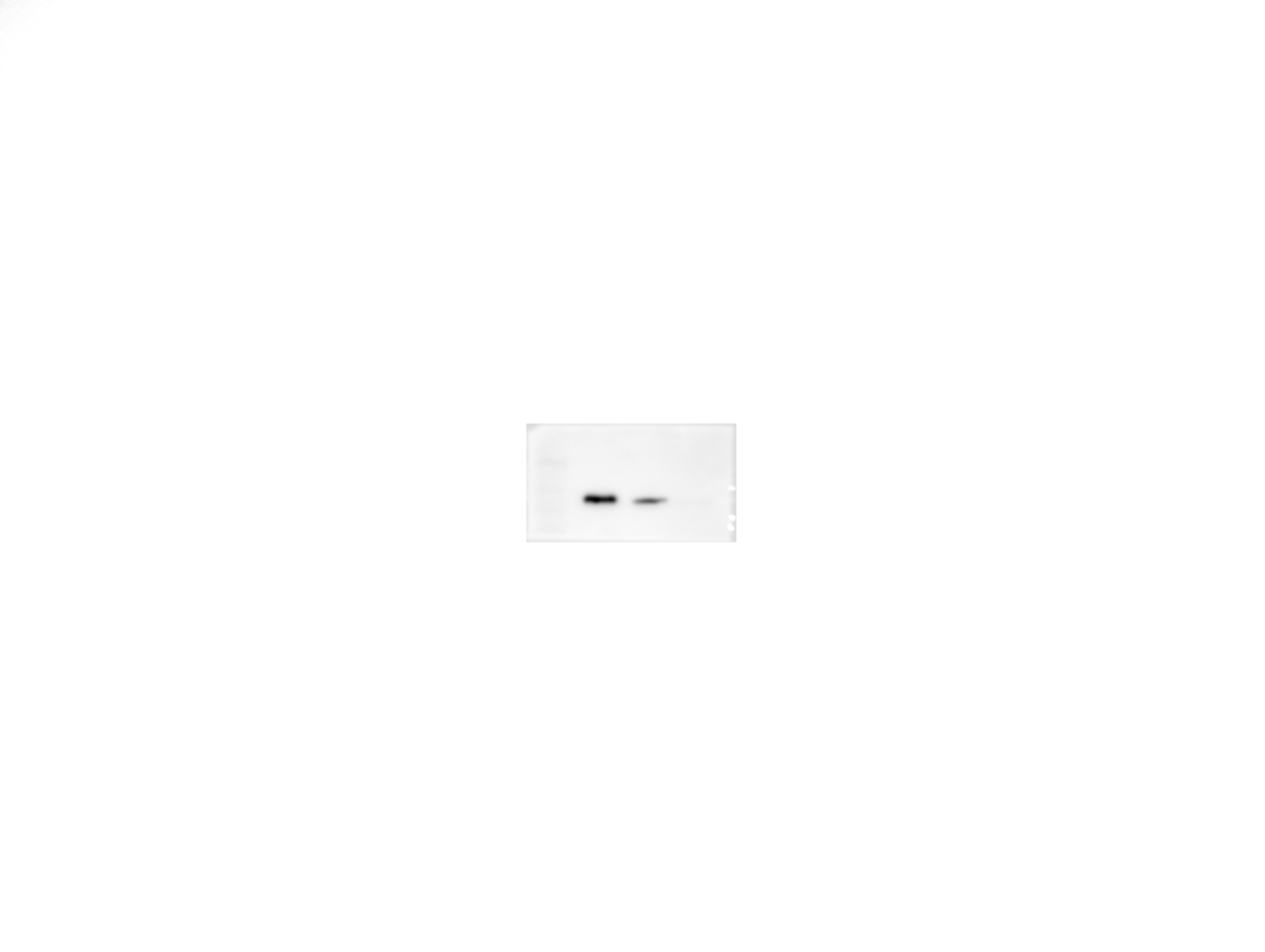

Supplement: Figure 2—source data 2. [file elife-100406-fig2-data2.zip › Figure 2-source data 2/F2-F-K562-IDH1.tif]

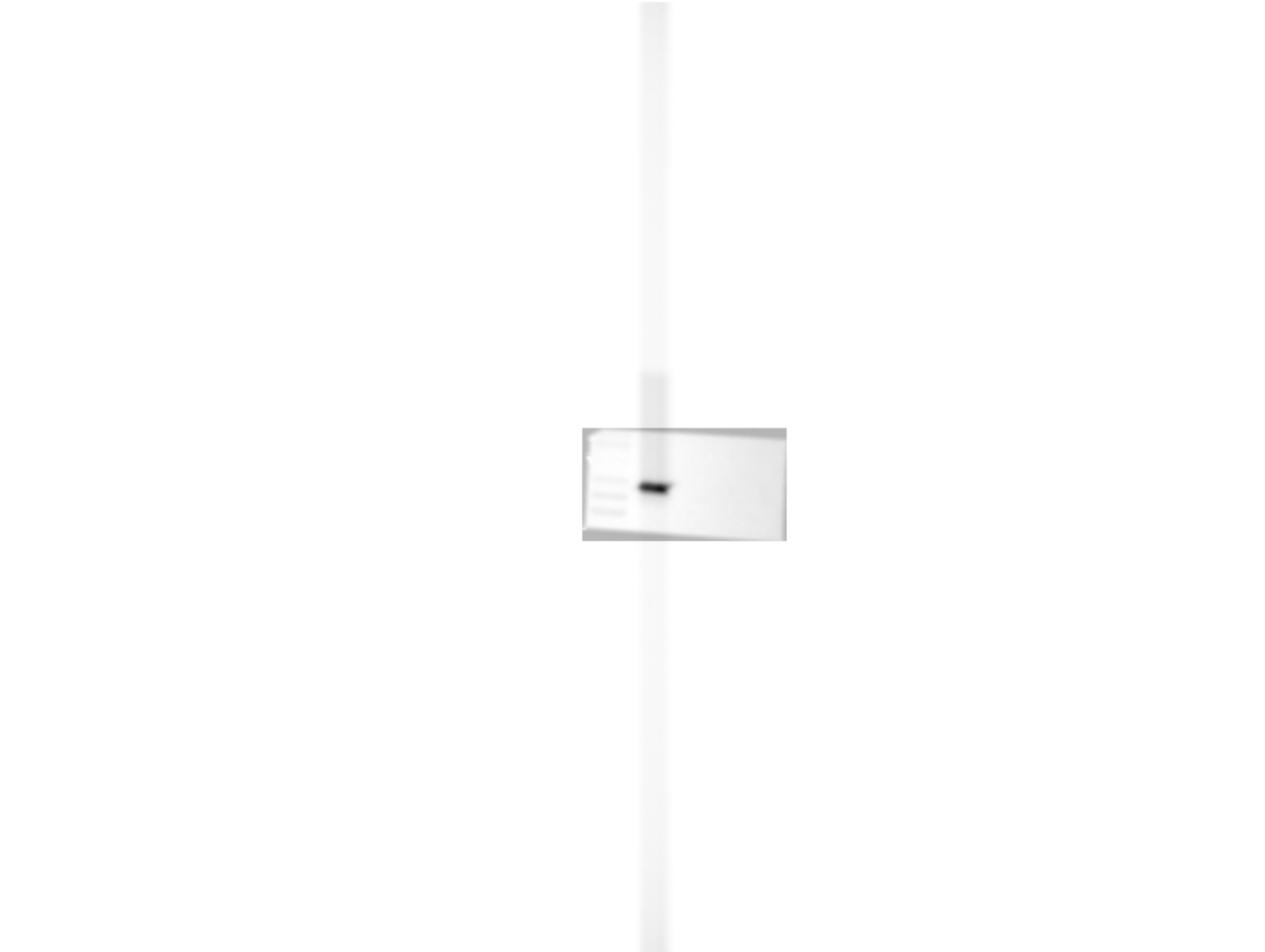

Supplement: Figure 2—source data 2. [file elife-100406-fig2-data2.zip › Figure 2-source data 2/F2-F-K562-RCC1.tif]

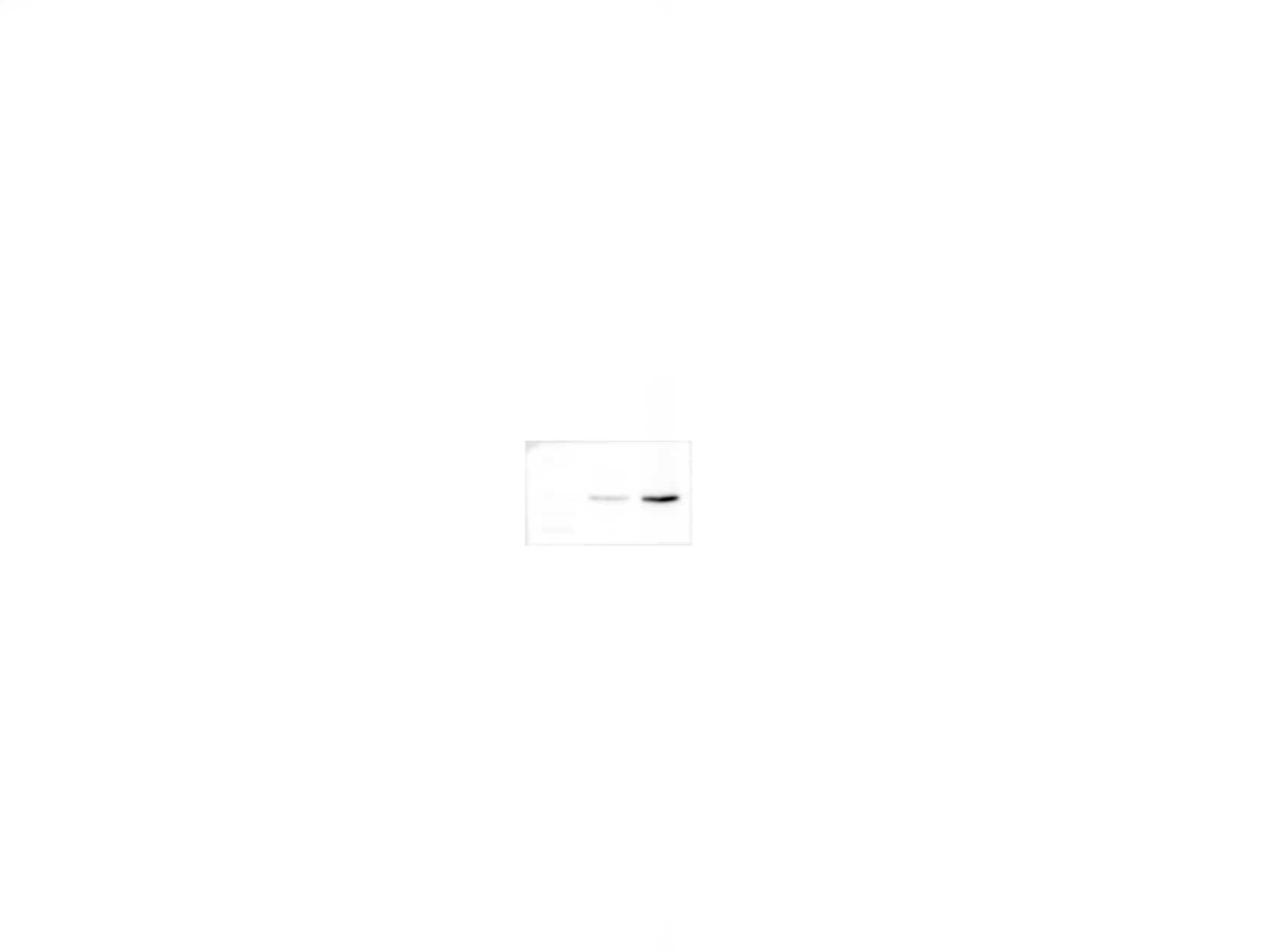

Supplement: Figure 2—source data 2. [file elife-100406-fig2-data2.zip › Figure 2-source data 2/F2-F-K562-Tubulin.tif]

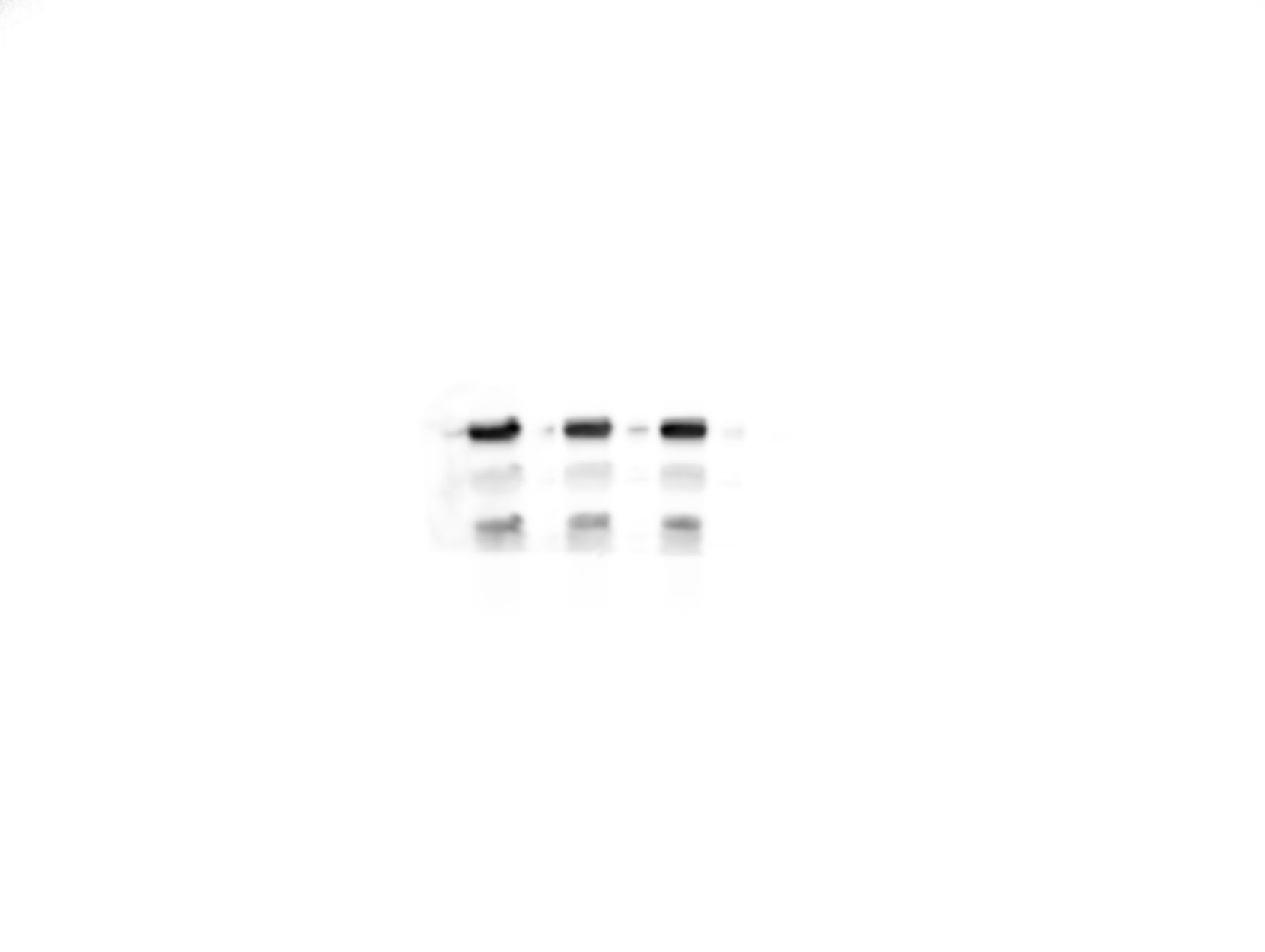

Supplement: Figure 2—source data 2. [file elife-100406-fig2-data2.zip › Figure 2-source data 2/F2-F-RCC1.tif]

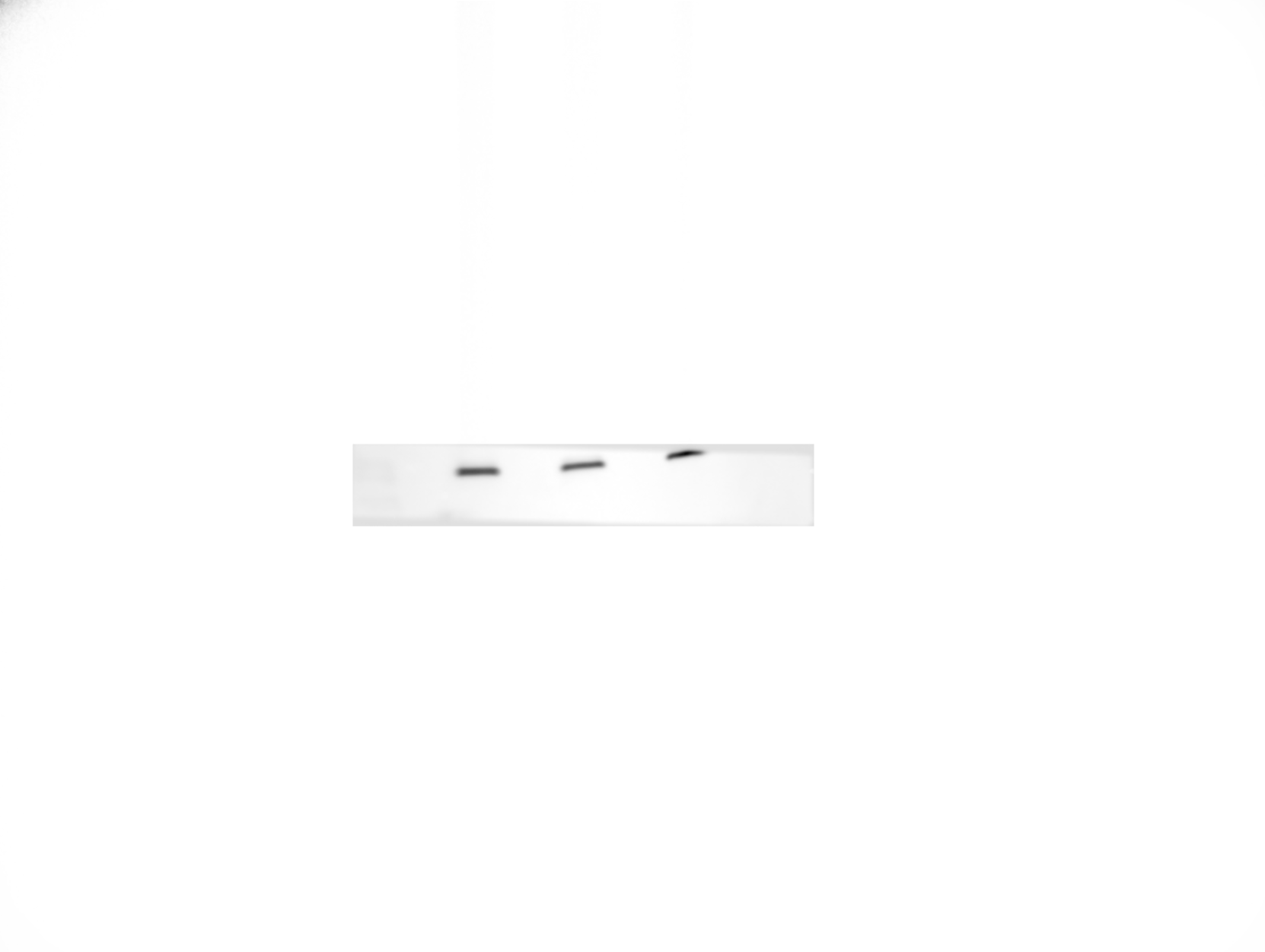

Supplement: Figure 2—source data 2. [file elife-100406-fig2-data2.zip › Figure 2-source data 2/F2-Tubulin.tif]

Figure 3D

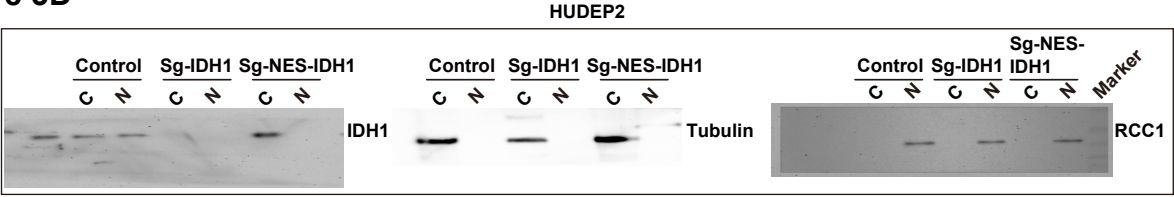

Supplement: Figure 3—source data 1. [file elife-100406-fig3-data1.pdf]

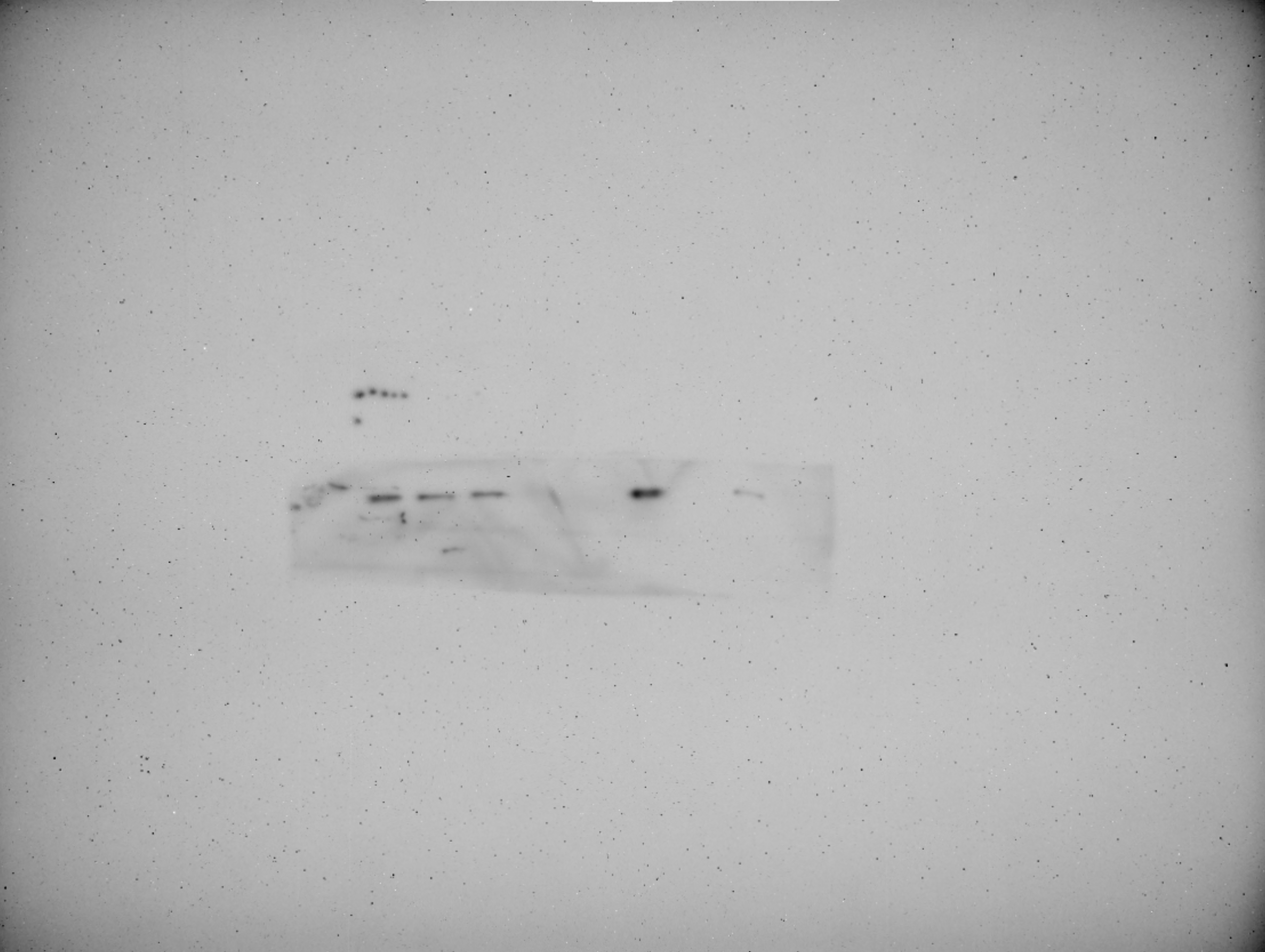

Supplement: Figure 3—source data 2. [file elife-100406-fig3-data2.zip › Figure 3-source data 2/F3-D-IDH1.tif]

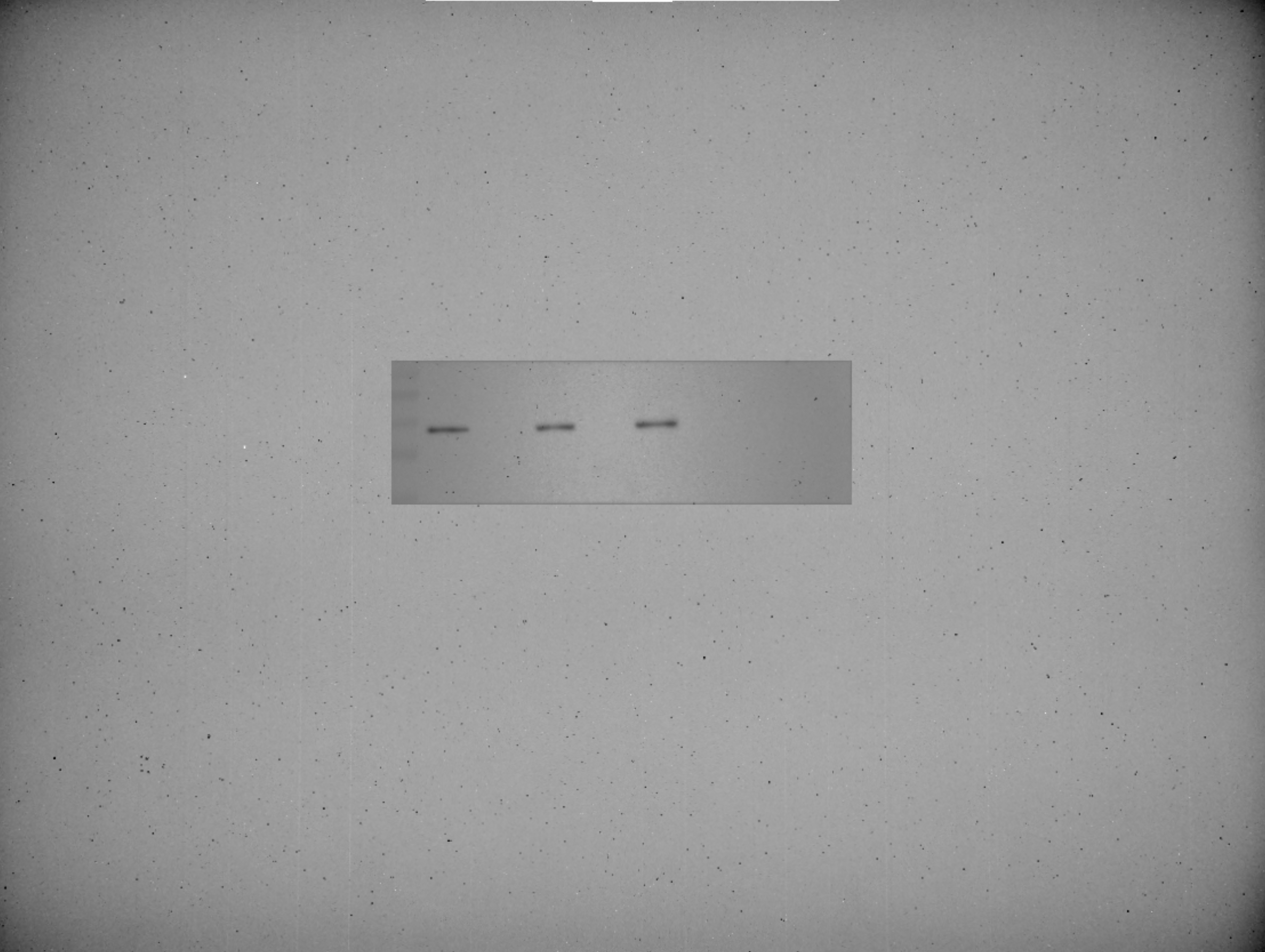

Supplement: Figure 3—source data 2. [file elife-100406-fig3-data2.zip › Figure 3-source data 2/F3-D-RCC1.tif]

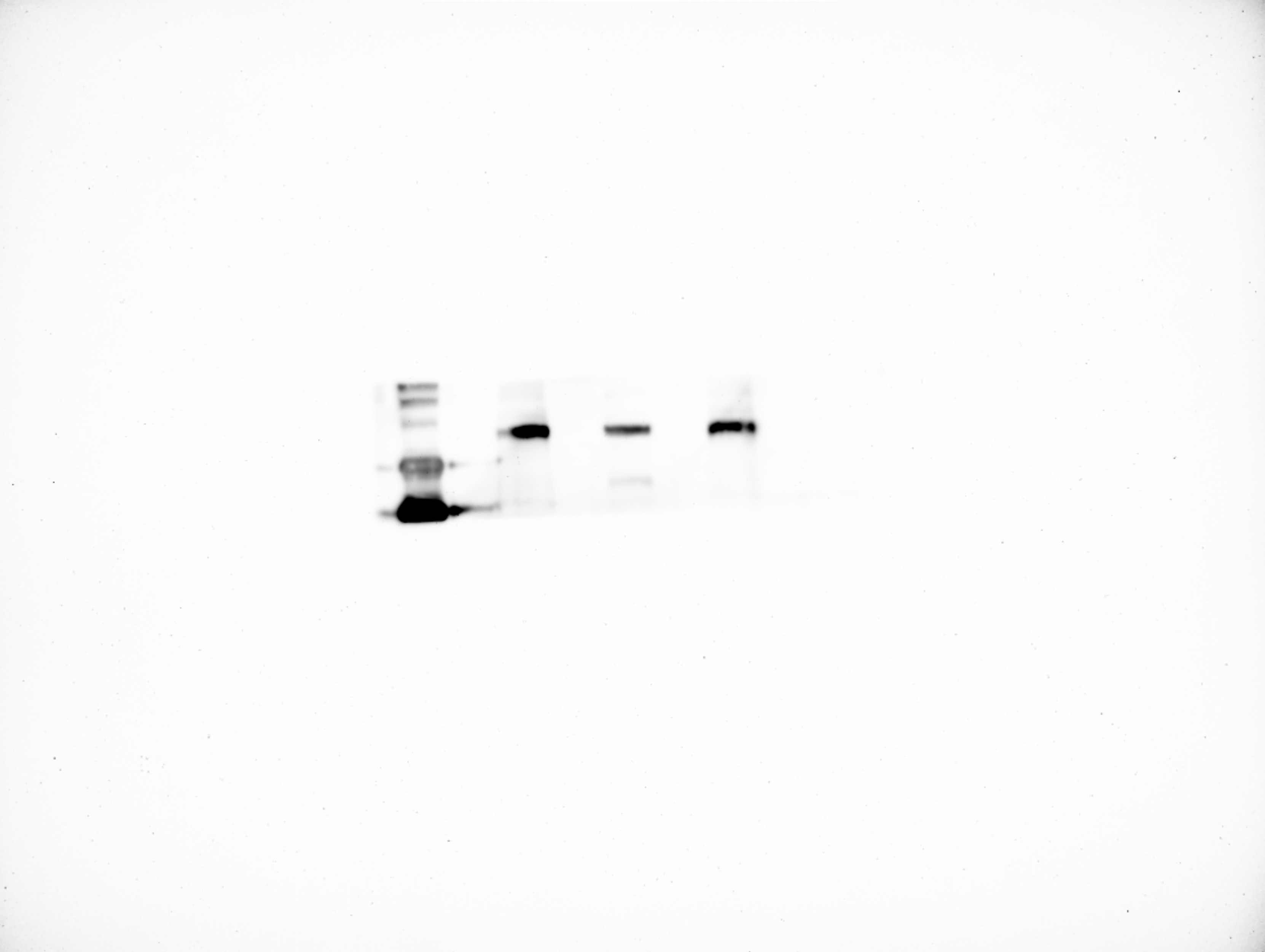

Supplement: Figure 3—source data 2. [file elife-100406-fig3-data2.zip › Figure 3-source data 2/F3-D-Tbulin.tif]

Figure 4A

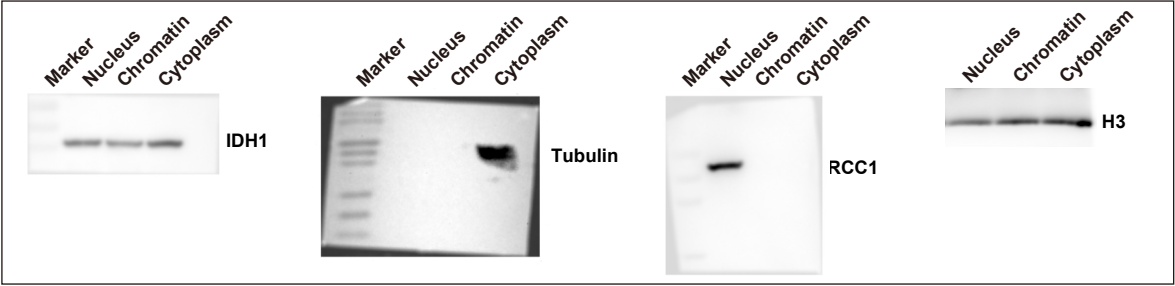

Figure 4E

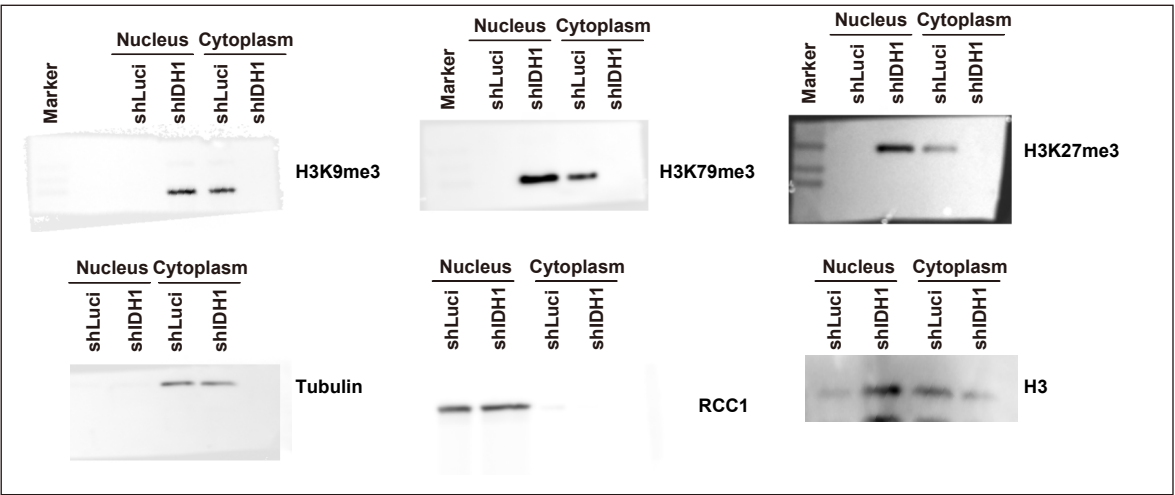

Supplement: Figure 4—source data 1. [file elife-100406-fig4-data1.pdf]

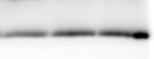

Supplement: Figure 4—source data 2. [file elife-100406-fig4-data2.zip › Figure 4-source data 2/F4-A-H3.tif]

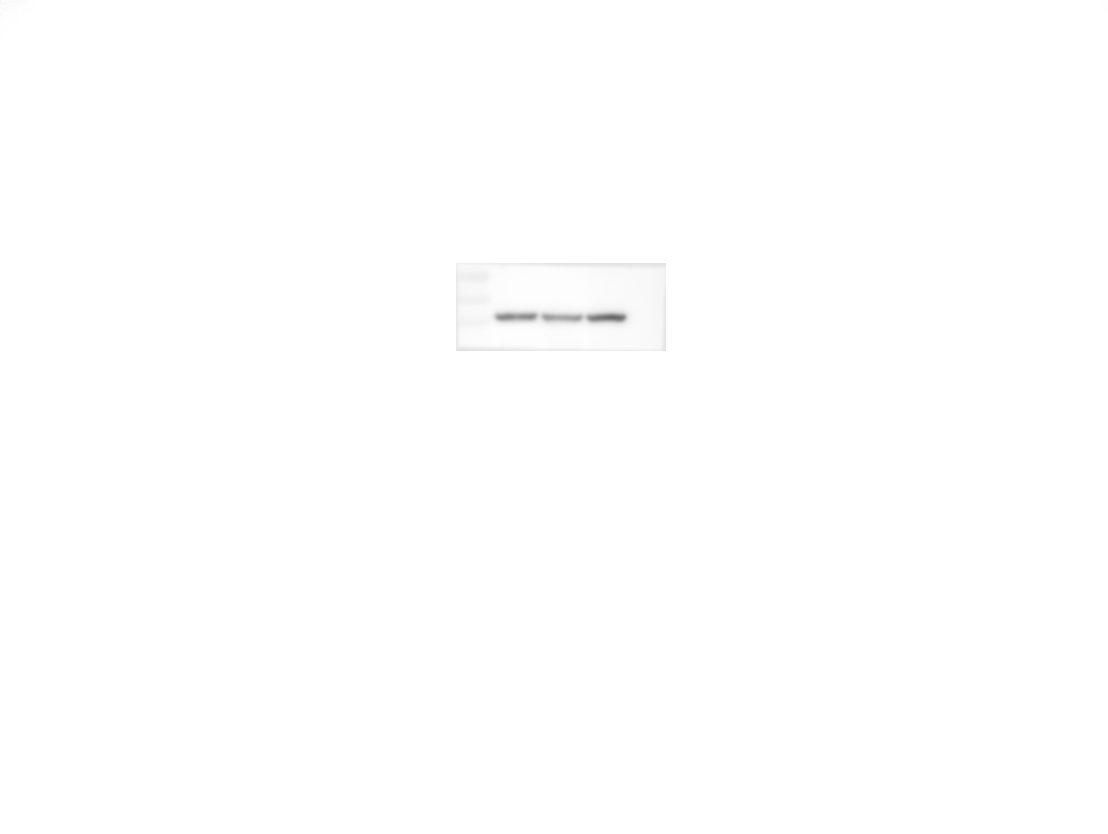

Supplement: Figure 4—source data 2. [file elife-100406-fig4-data2.zip › Figure 4-source data 2/F4-A-IDH1.tif]

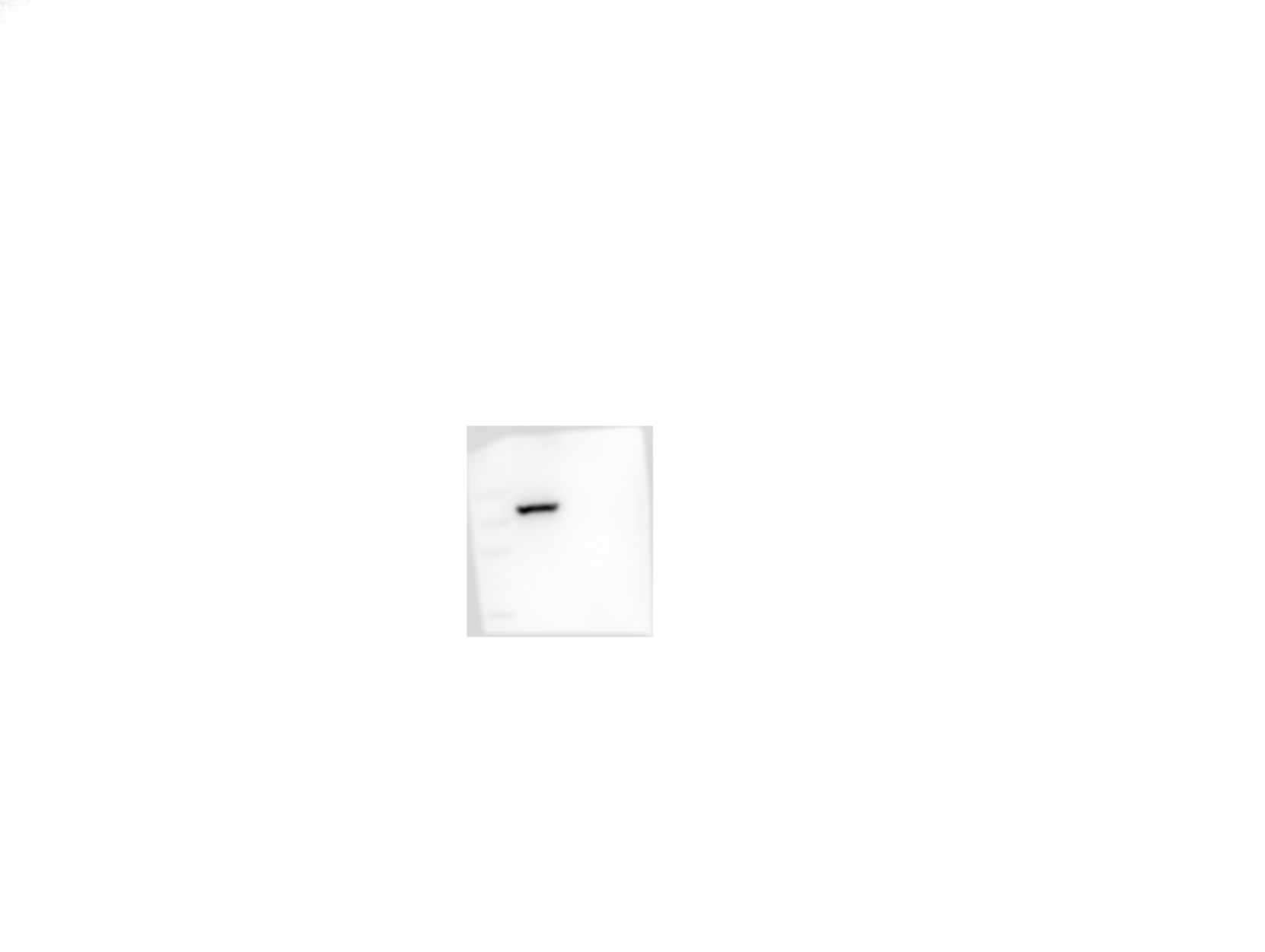

Supplement: Figure 4—source data 2. [file elife-100406-fig4-data2.zip › Figure 4-source data 2/F4-A-RCC1.tif]

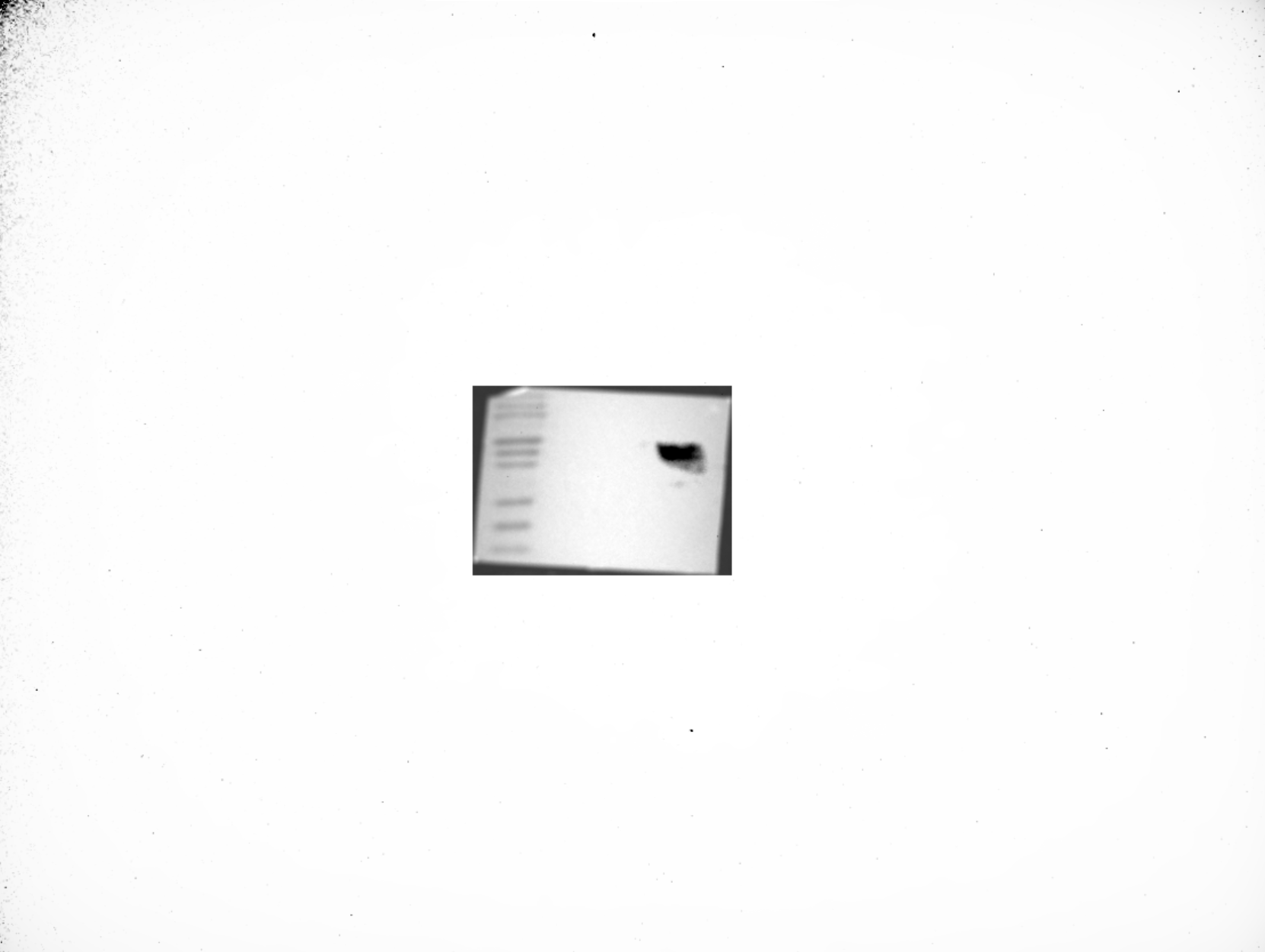

Supplement: Figure 4—source data 2. [file elife-100406-fig4-data2.zip › Figure 4-source data 2/F4-A-Tubulin.tif]

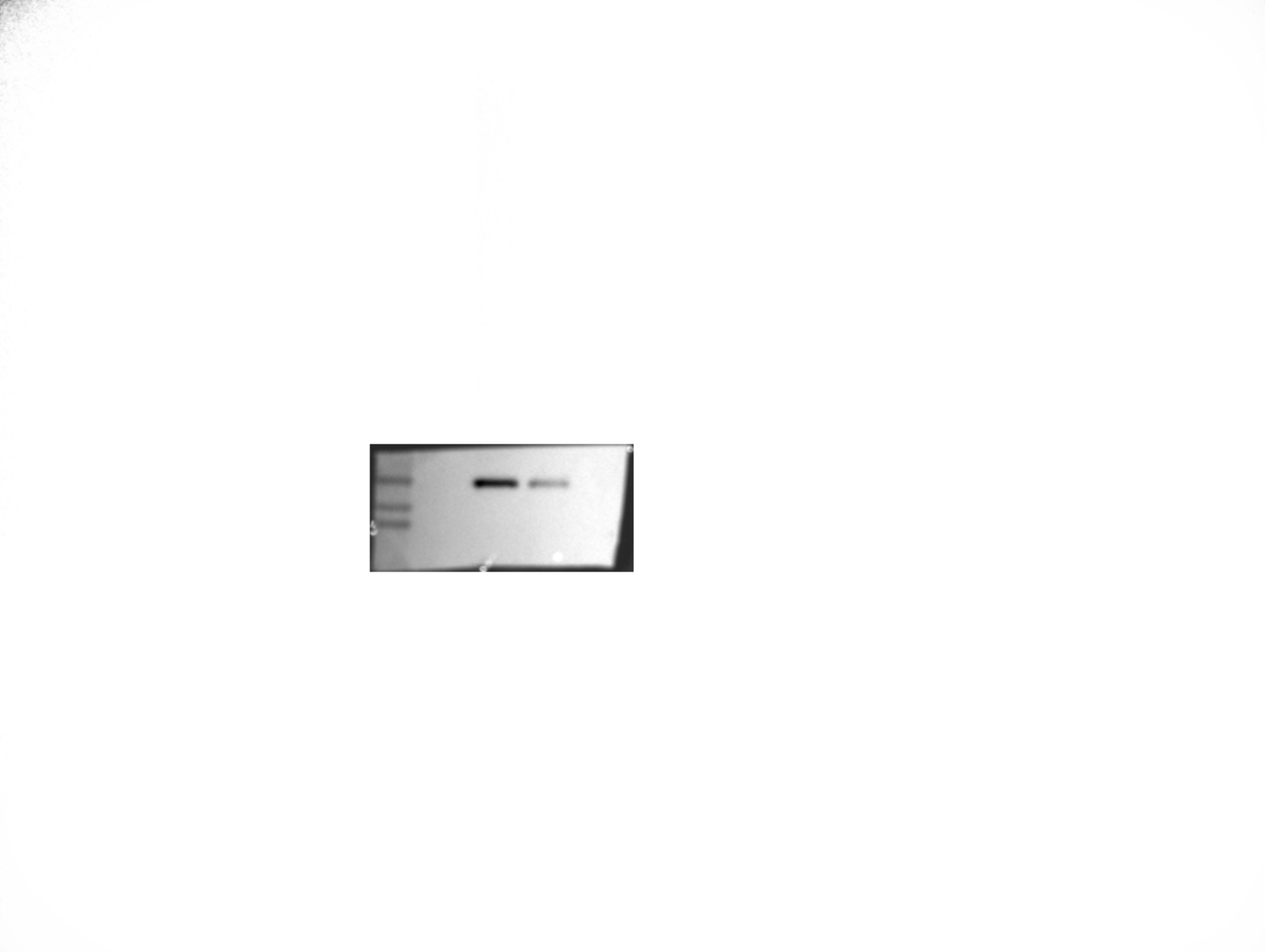

Supplement: Figure 4—source data 2. [file elife-100406-fig4-data2.zip › Figure 4-source data 2/F4-E-H3K27me2.tif]

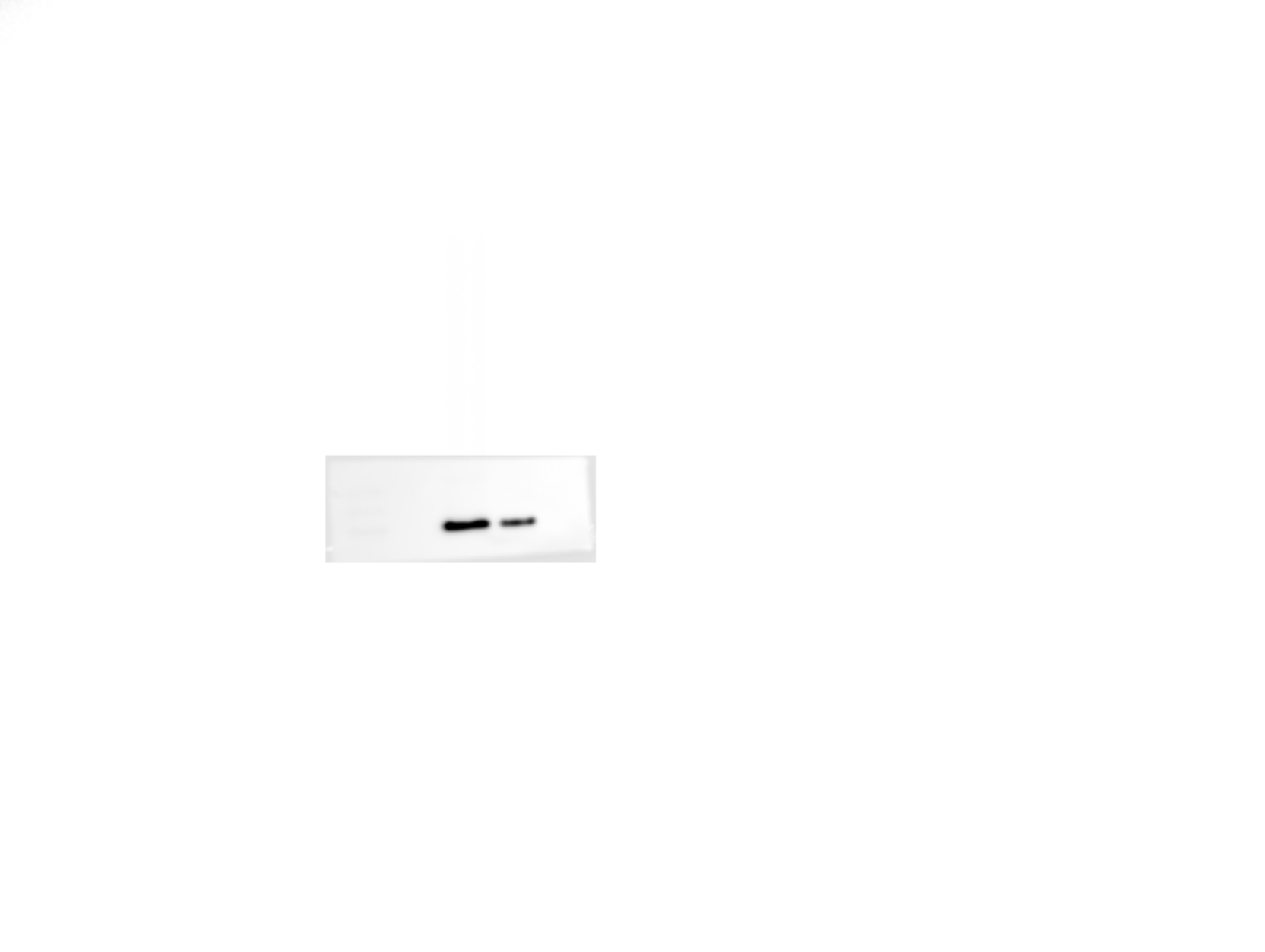

Supplement: Figure 4—source data 2. [file elife-100406-fig4-data2.zip › Figure 4-source data 2/F4-E-H3K79me3.tif]

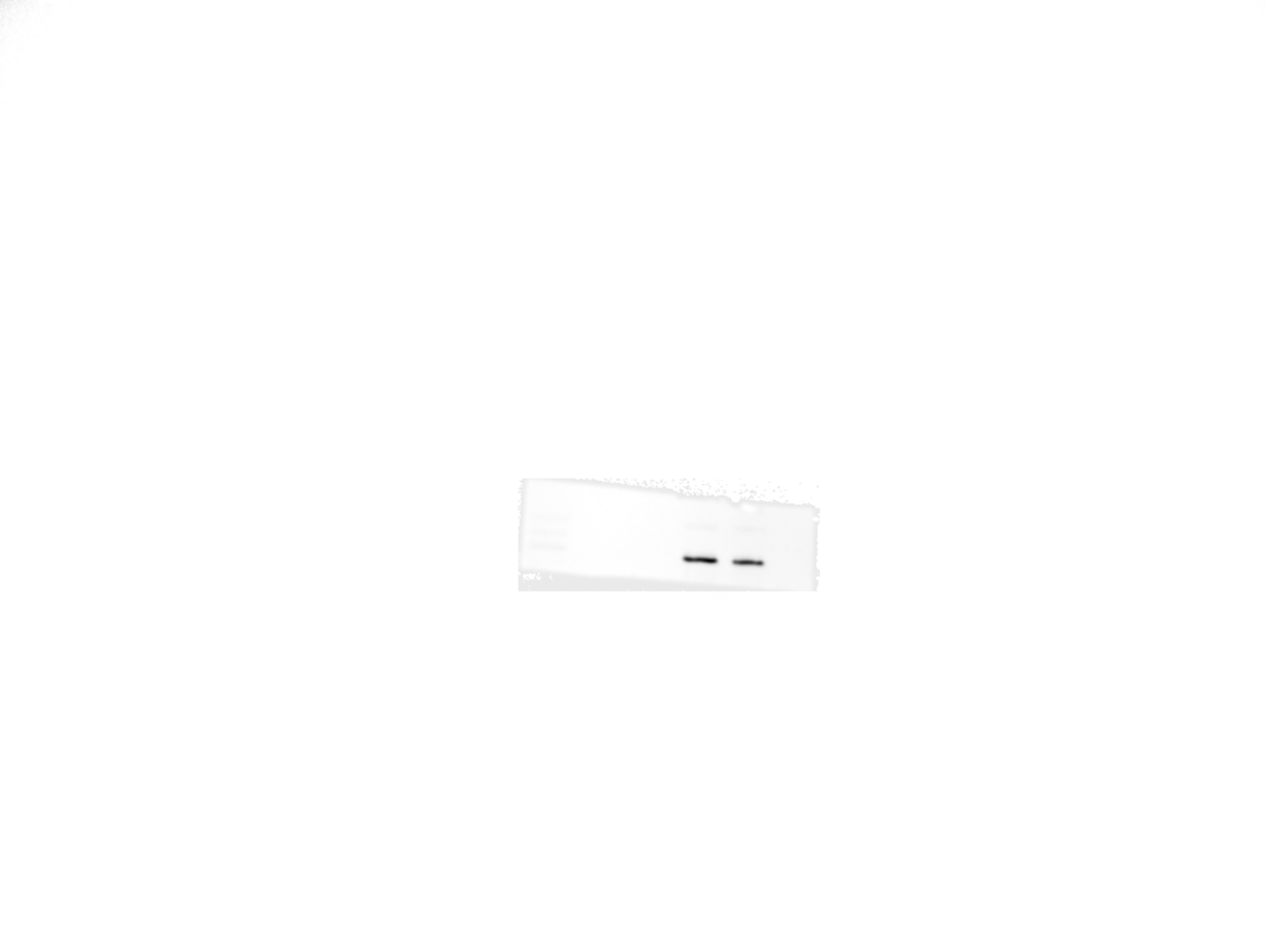

Supplement: Figure 4—source data 2. [file elife-100406-fig4-data2.zip › Figure 4-source data 2/F4-E-H3K9me3.tif]

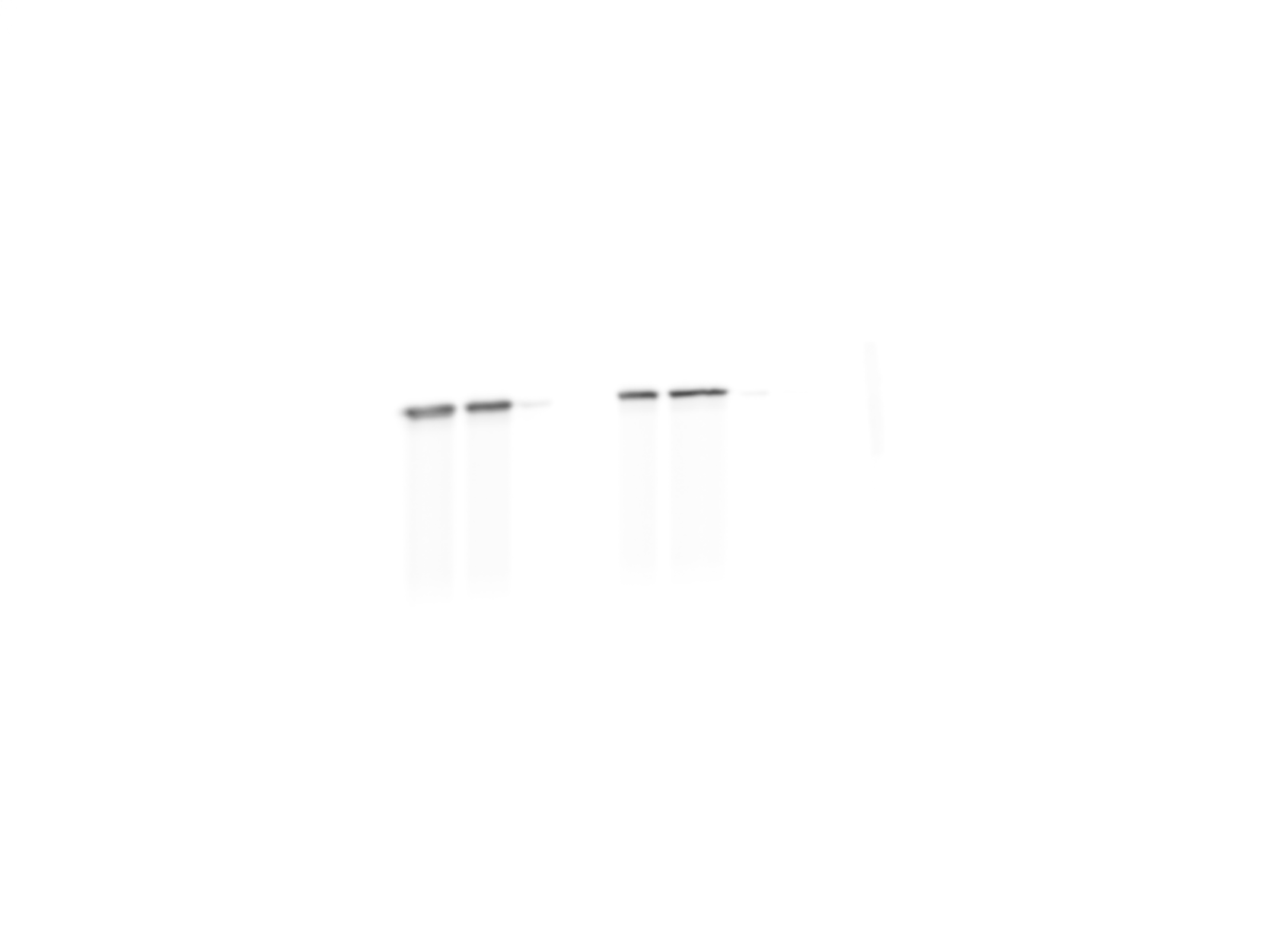

Supplement: Figure 4—source data 2. [file elife-100406-fig4-data2.zip › Figure 4-source data 2/F4-E-RCC1.tif]

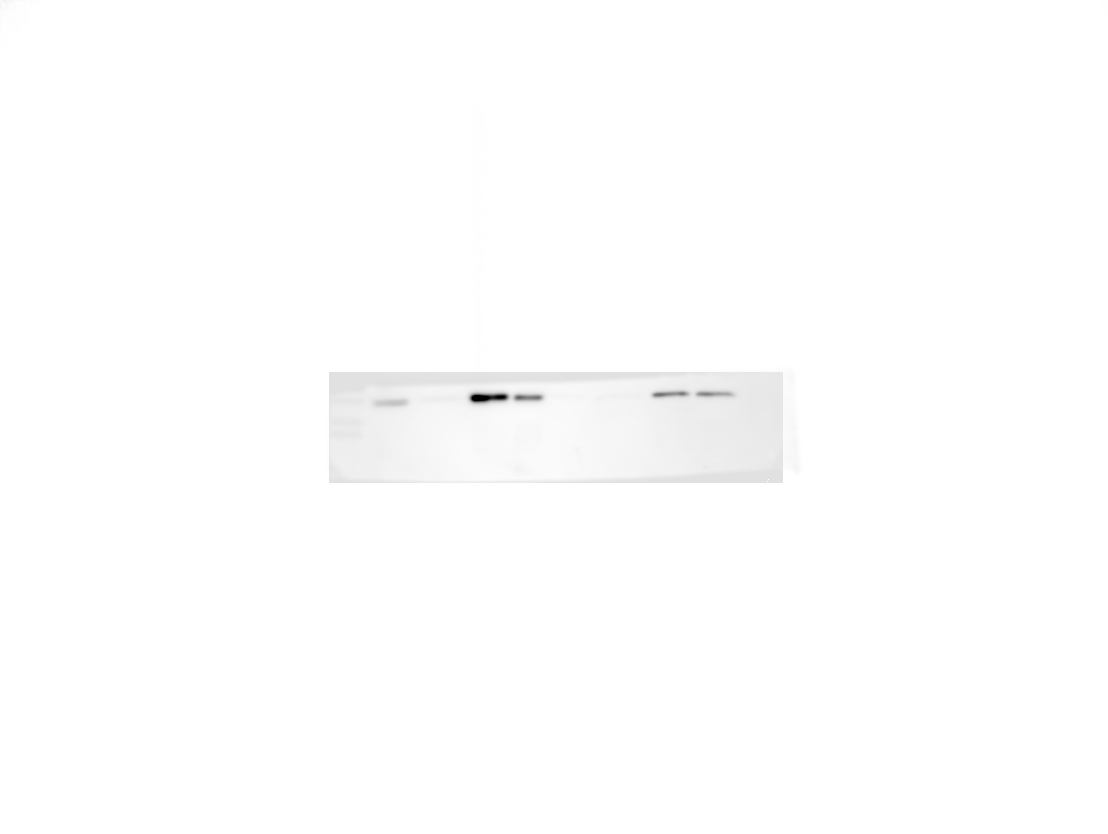

Supplement: Figure 4—source data 2. [file elife-100406-fig4-data2.zip › Figure 4-source data 2/F4-E-tubulin.tif]

Figure S10A

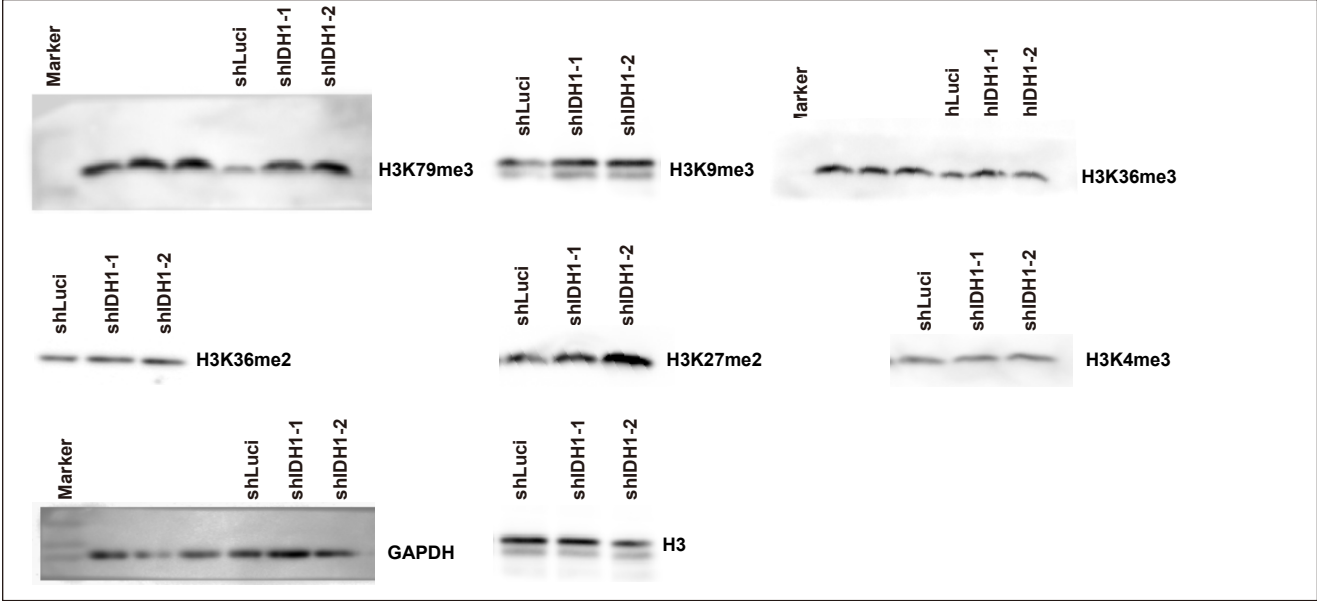

Supplement: Figure 4—figure supplement 1—source data 1. [file elife-100406-fig4-figsupp1-data1.pdf]

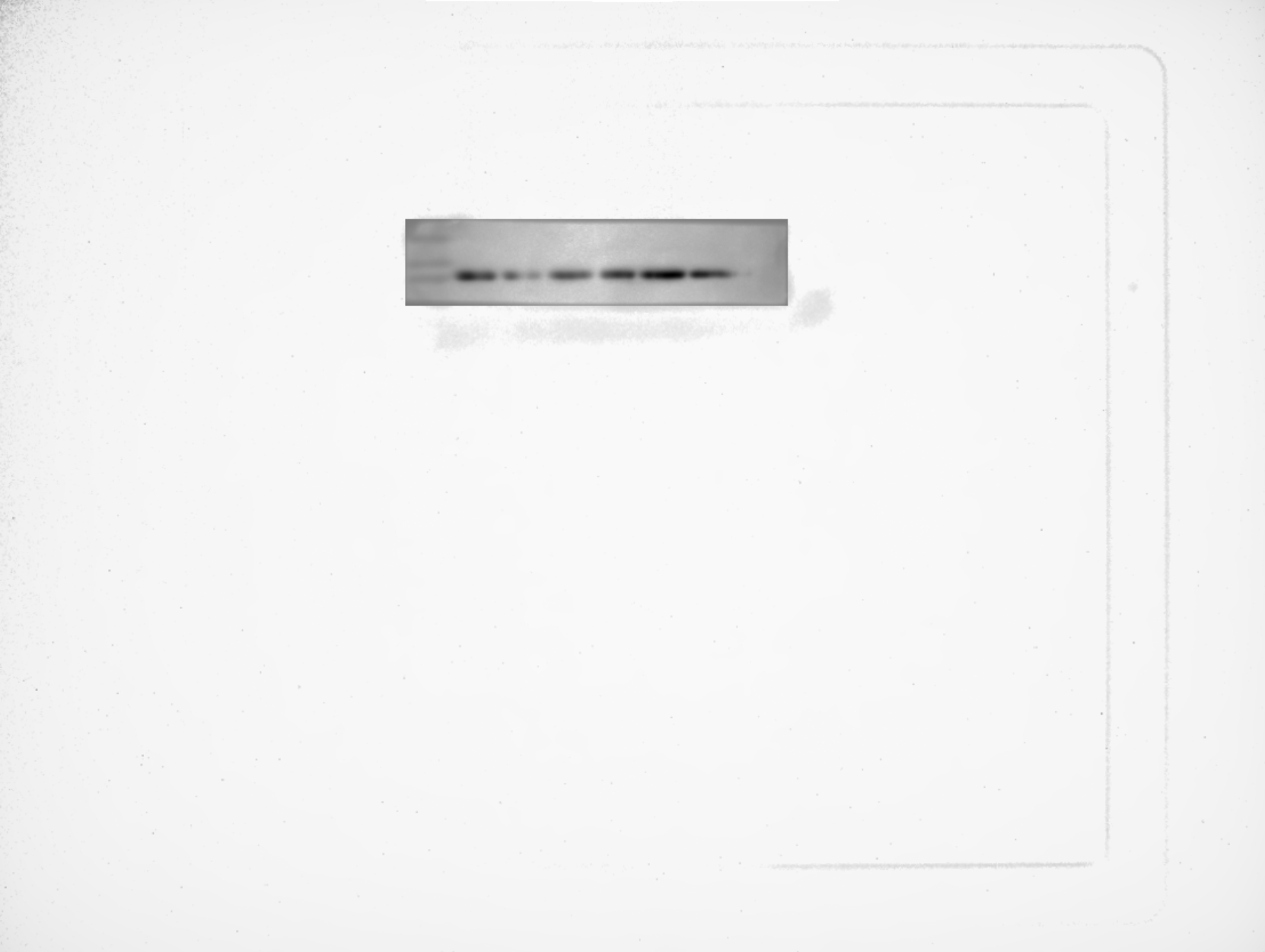

Supplement: Figure 4—figure supplement 1—source data 2. [file elife-100406-fig4-figsupp1-data2.zip › Figure S10-source data 2/SF10-A-gapdh.tif]

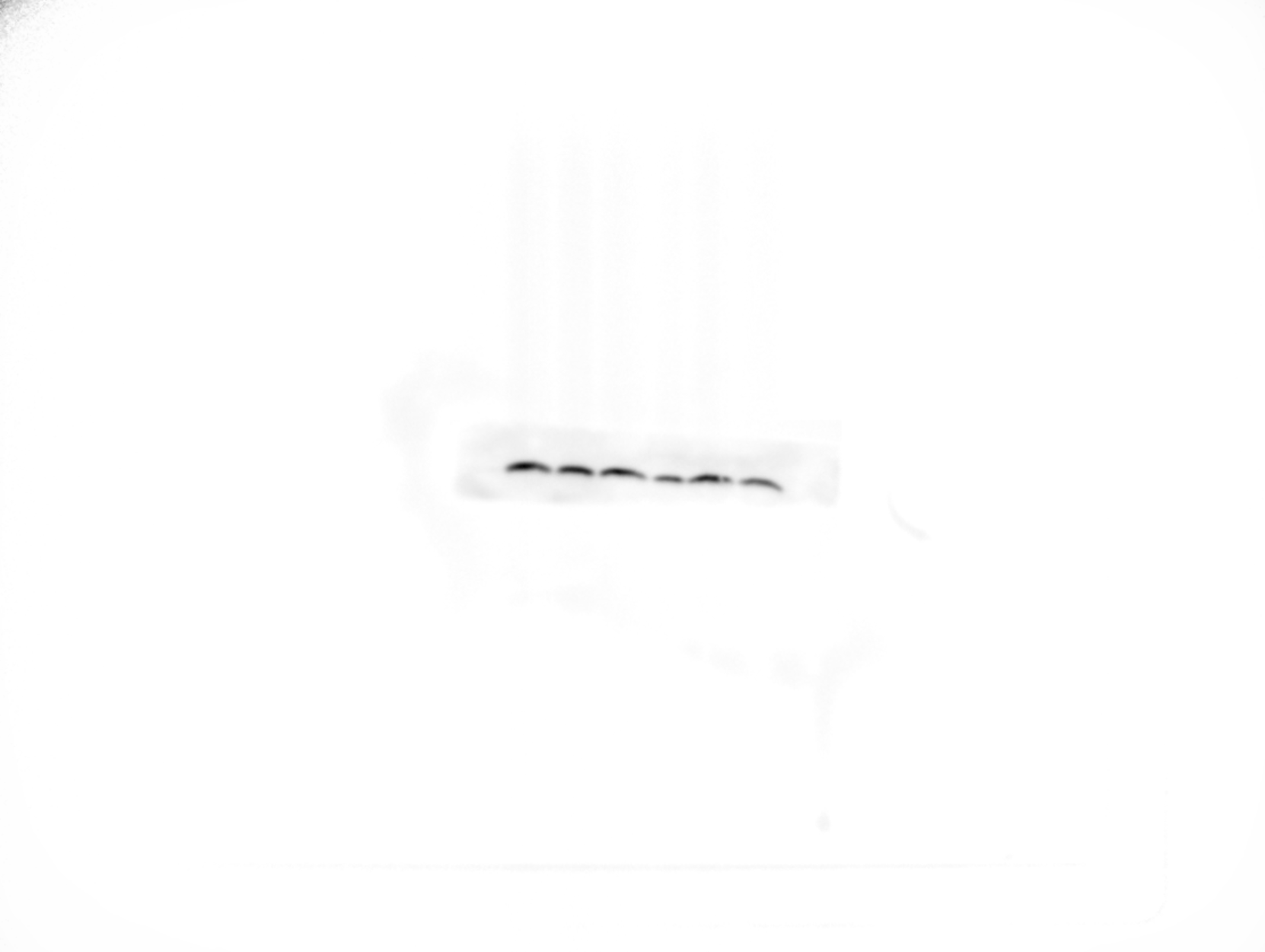

Supplement: Figure 4—figure supplement 1—source data 2. [file elife-100406-fig4-figsupp1-data2.zip › Figure S10-source data 2/SF10-A-H3K36me3.tif]

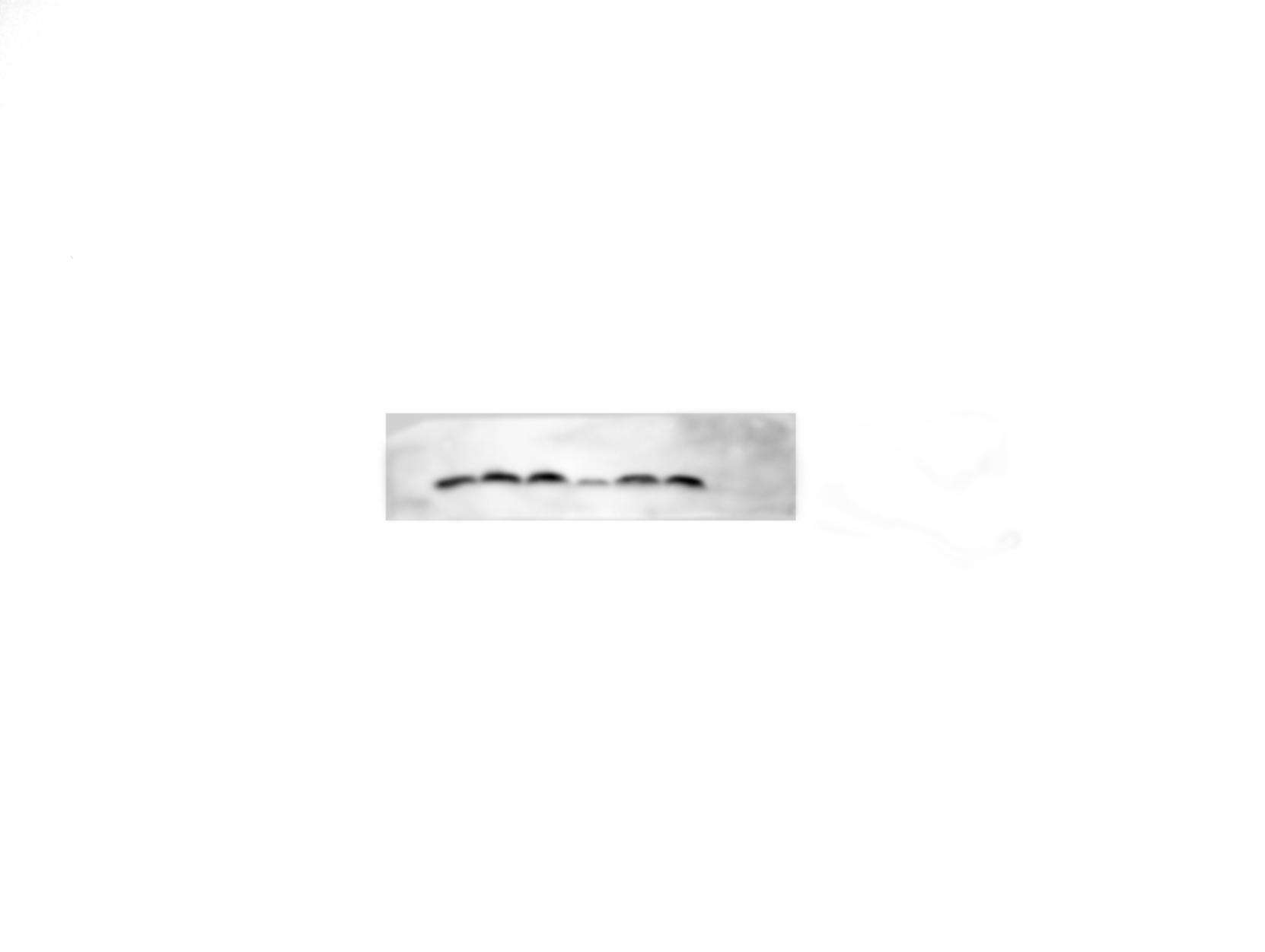

Supplement: Figure 4—figure supplement 1—source data 2. [file elife-100406-fig4-figsupp1-data2.zip › Figure S10-source data 2/SF10-A-H3K79me3.tif]

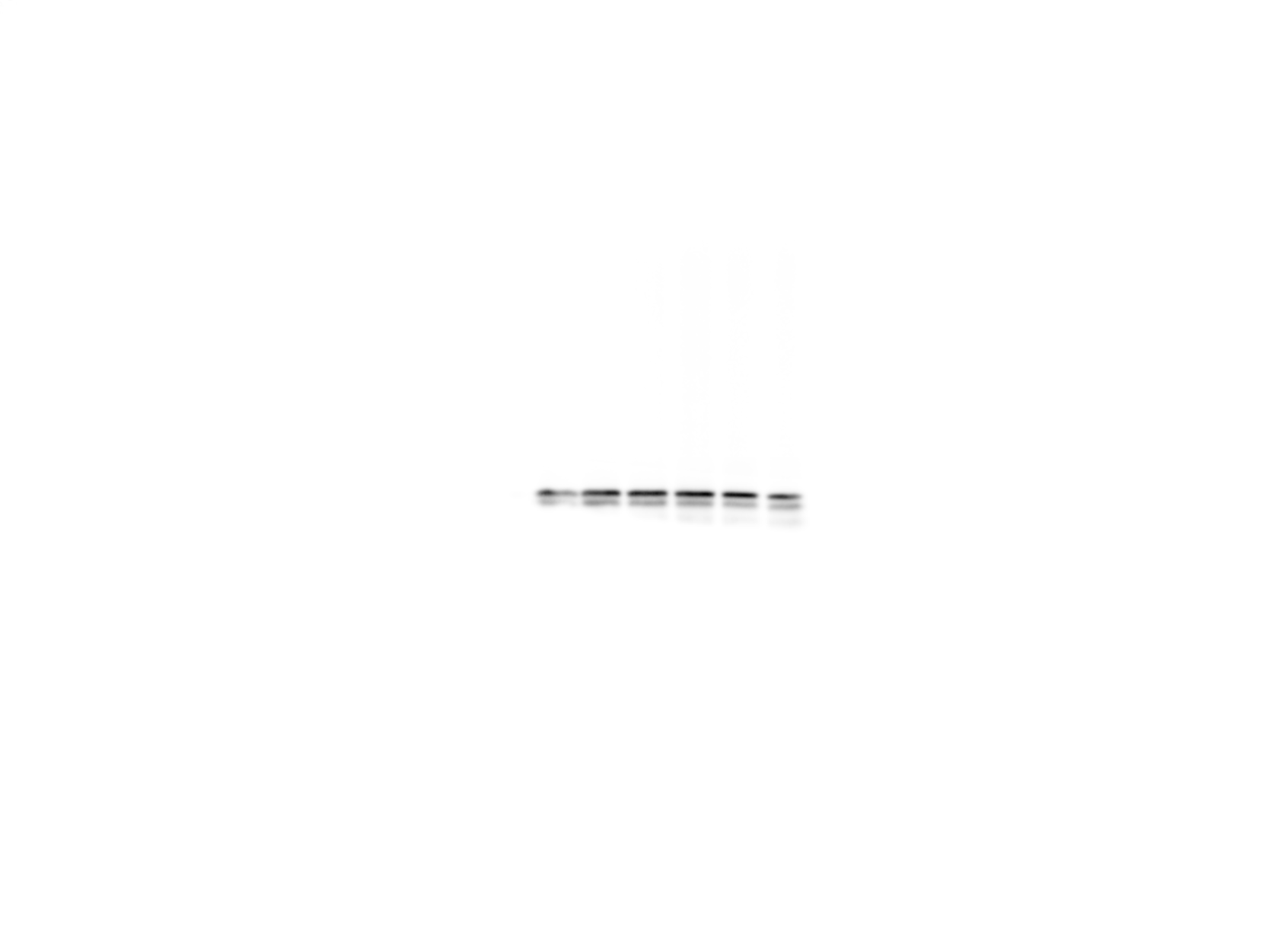

Supplement: Figure 4—figure supplement 1—source data 2. [file elife-100406-fig4-figsupp1-data2.zip › Figure S10-source data 2/SF10-A-H3K9me3-H3.tif]

Figure 7F

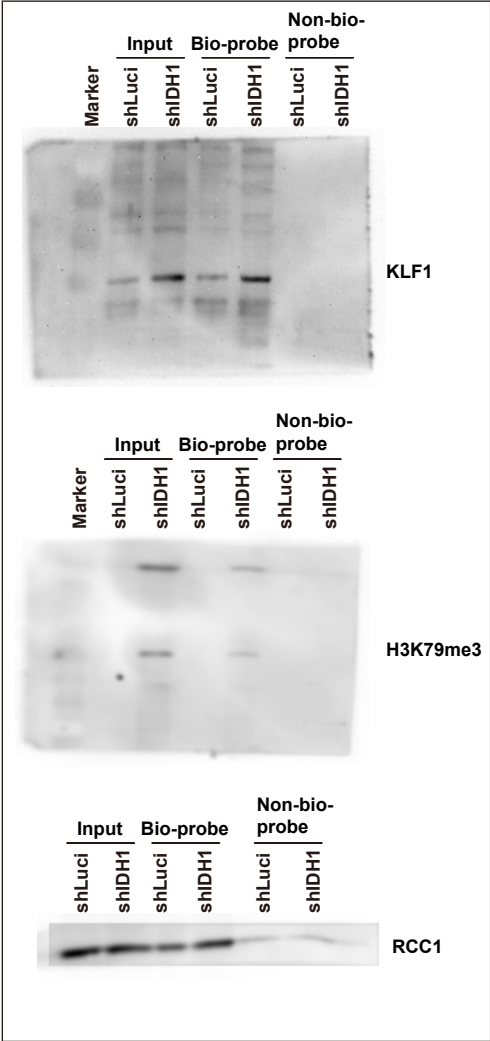

Supplement: Figure 7—source data 1. [file elife-100406-fig7-data1.pdf]

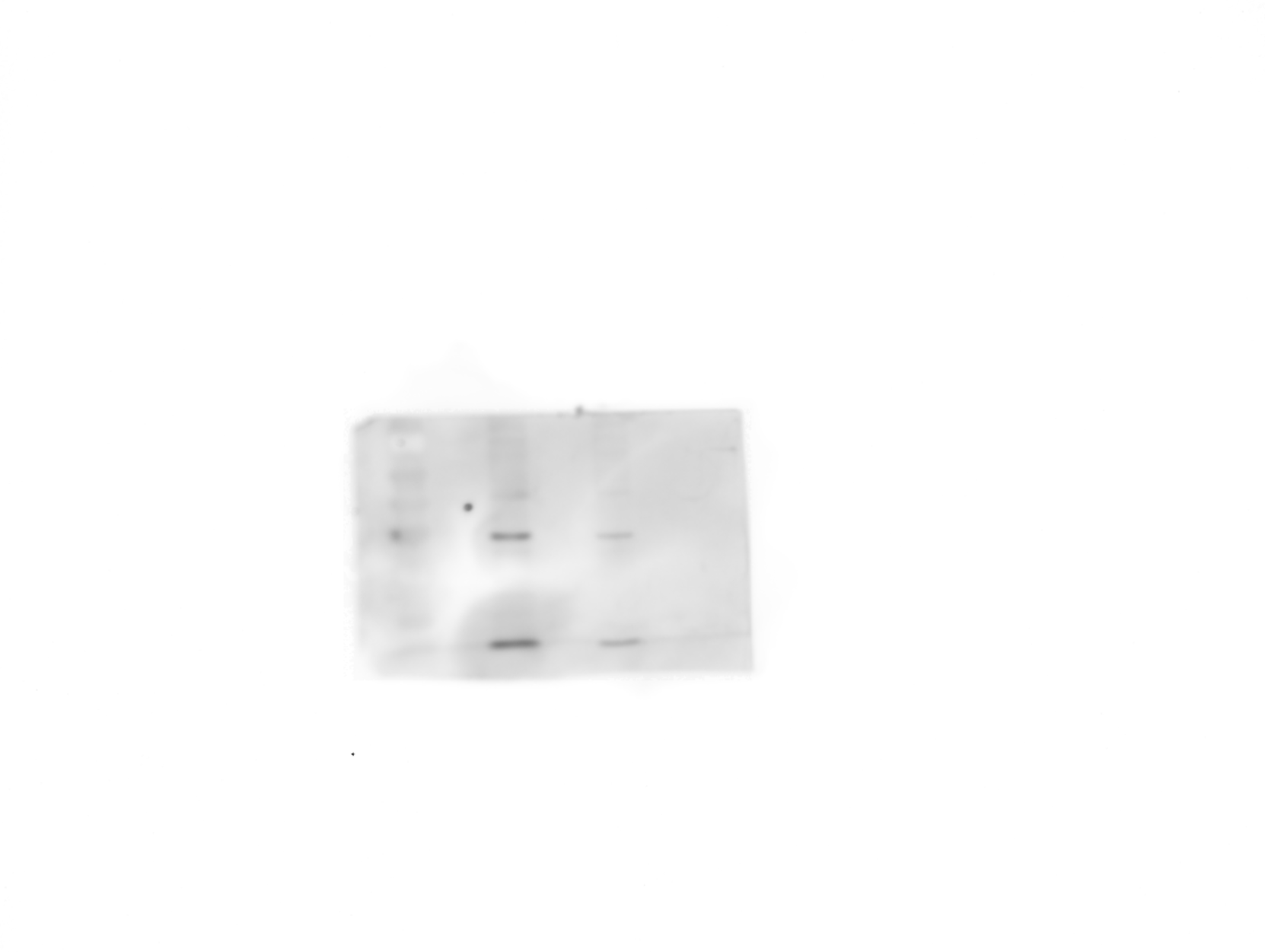

Supplement: Figure 7—source data 2. [file elife-100406-fig7-data2.zip › Figure 7-source data 2/F7-F-H3K79me3.tif]

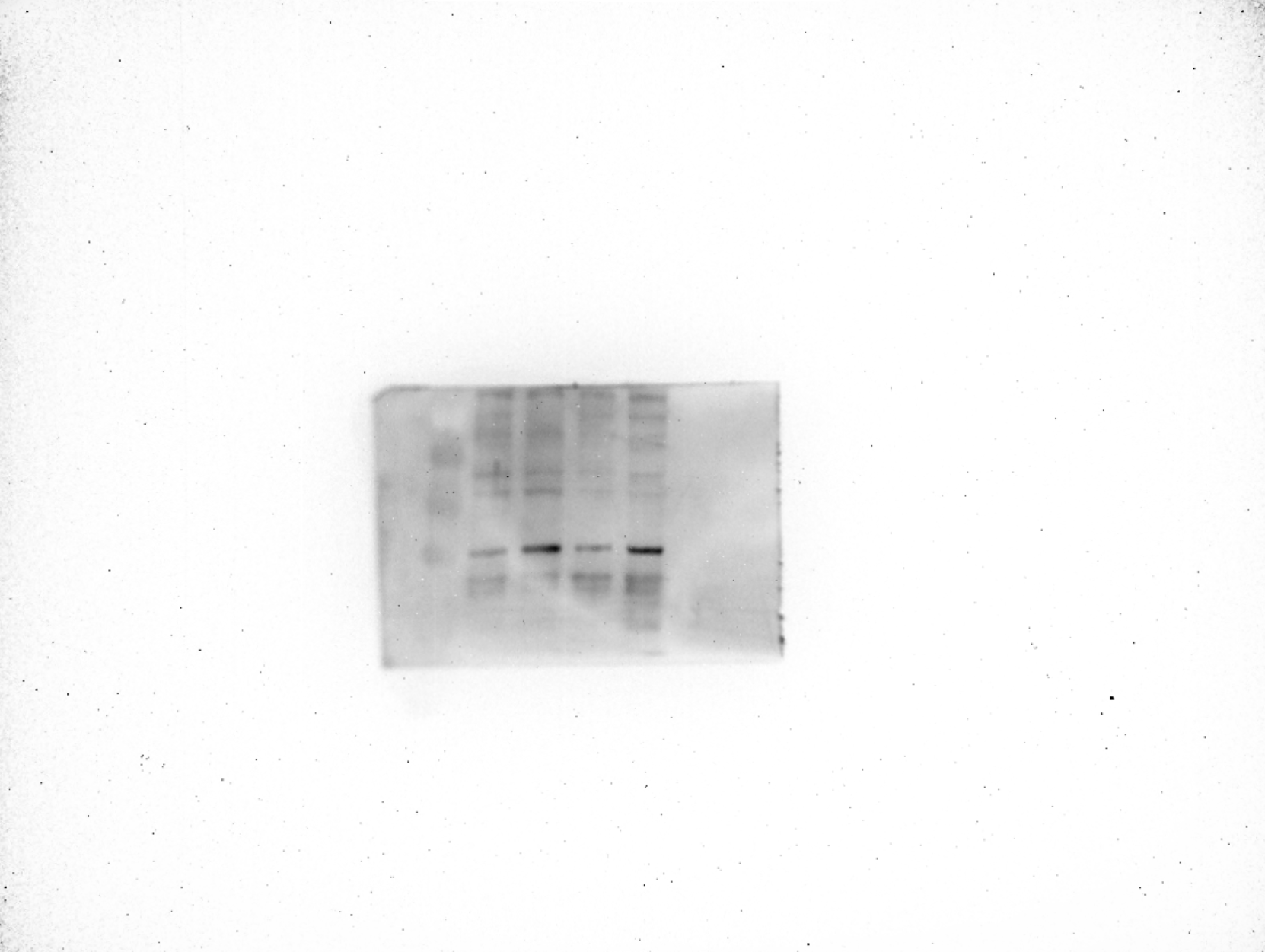

Supplement: Figure 7—source data 2. [file elife-100406-fig7-data2.zip › Figure 7-source data 2/F7-F-KLF1.tif]

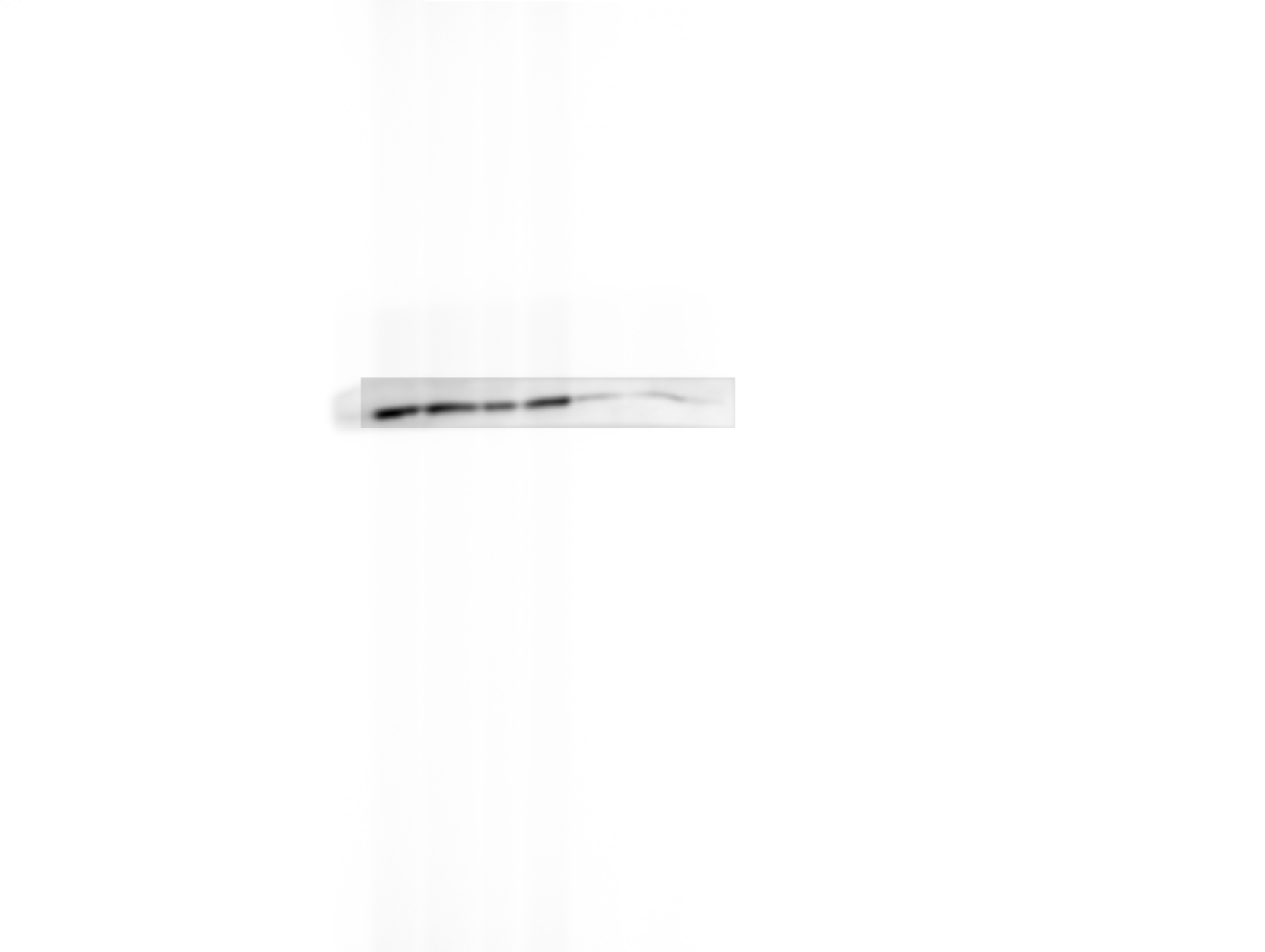

Supplement: Figure 7—source data 2. [file elife-100406-fig7-data2.zip › Figure 7-source data 2/F7-F-RCC1.tif]
