# Supplementary material for: IDH1 regulates human erythropoiesis by eliciting chromatin state reprogramming
Source: eLife. 2025 Apr 29;13:RP100406. doi: 10.7554/eLife.100406 (PMC12040319; doi:10.7554/eLife.100406)
Supplement: Supplementary file 1. [file elife-100406-supp1.docx]

**Table 1. Information for Human Research Participants**

| **No.** | **Sex** | **Age** | **Disease Type** | **Mutant Site** | **Frequency (%)** |
| --- | --- | --- | --- | --- | --- |
| 1 | woman | 71 | AML | IDH1:NM_005896:exon4:c.C394T:p.R132C rs121913499 | 45.9 |
| 2 | woman | 16 | AML | IDH1:NM_005896:exon4:c.C394T:p.R132C rs121913499 | 45.81 |
| 3 | woman | 64 | MDS-EB1 | IDH1:NM_005896:exon4:c.C394T:p.R132C rs121913499 | 29.86 |
| 4 | woman | 48 | AML | IDH1:NM_005896:exon4:c.C394G:p.R132G rs121913499 | 26.98 |
| 5 | woman | 55 | AML | IDH1:NM_005896:exon4:c.C394T:p.R132C rs121913499 | 25.47 |
| 6 | woman | 71 | MDS-RAEB2 | IDH1:NM_005896:exon4:c.C394T:p.R132C rs121913499 | 18.72 |
| 7 | man | 56 | AML | IDH1:NM_005896:exon4:c.C394T:p.R132C rs121913499 | 13.16 |
| 8 | woman | 75 | MDS | IDH1:NM_005896:exon4:c.C394T:p.R132C rs121913499 | 12.97 |
| 9 | man | 46 | MDS-EB2 | IDH1:NM_005896:exon4:c.G395A:p.R132H rs121913500 | 5.71 |
| 10 | man | 59 | AML | IDH1:NM_005896:exon4:c.G395A:p.R132H rs121913500 | 1.93 |

**Table 2. The sequence of IDH1-shRNA and IDH1-siRNA**

| **Primer** | **Sequence (5'-3')** |
| --- | --- |
| IDH1-shRNA1 | Forward: CCGGCCTATCATCATAGGTCGTCATCTCGAGATGACGACCTATGATGATAGGTTTTTG |
|  | Reverse: AATTCAAAAACCTATCATCATAGGTCGTCATCTCGAGATGACGACCTATGATGATAGG |
| IDH1-shRNA2 | Forward: CCGGCCTTTGTATCTGAGCACCAAACTCGAGTTTGGTGCTCAGATACAAAGGTTTTTG |
|  | Reverse: AATTCAAAAACCTTTGTATCTGAGCACCAACTCGAGTTTGGTGCTCAGATACAAAGG |
| IDH1-siRNA1 | GGCCCAAGCUAUGAAAUCATT |
| IDH1-siRNA2 | CCUGGUACAUAACUUUGAATT |

**Table 3. qPCR primer sequences of IDH1 and GAPDH**

| **Primer** | **Sequence (5'-3')** |
| --- | --- |
| IDH1 | Forward: TGTGGTAGAGATGCAAGGAGA |
|  | Reverse: TTGGTGACTTGGTTGGTG |
| GAPDH | Forward: CATGAGAAGTATGACAACAGCCT |
|  | Reverse: AGTCCTTCCACGATACCAAAGT |
